# Structures of monomeric and dimeric PRC2:EZH1 reveal flexible modules involved in chromatin compaction

**DOI:** 10.1038/s41467-020-20775-z

**Published:** 2021-01-29

**Authors:** Daniel Grau, Yixiao Zhang, Chul-Hwan Lee, Marco Valencia-Sánchez, Jenny Zhang, Miao Wang, Marlene Holder, Vladimir Svetlov, Dongyan Tan, Evgeny Nudler, Danny Reinberg, Thomas Walz, Karim-Jean Armache

**Affiliations:** 1 Skirball Institute of Biomolecular Medicine, Department of Biochemistry and Molecular Pharmacology, New York University Grossman School of Medicine, New York, NY USA; 2 Laboratory of Molecular Electron Microscopy, The Rockefeller University, New York, NY USA; 3 Department of Biochemistry and Molecular Pharmacology, New York University Grossman School of Medicine, New York, NY USA; 4 Howard Hughes Medical Institute, Chevy Chase, MD USA; 5 Department of Pharmacological Sciences, Stony Brook University Medical School, Stony Brook, NY USA; 6 Present Address: Department of Pharmacology, Seoul National University, Seoul, Republic of Korea

**Keywords:** Chromatin structure, Histone post-translational modifications

## Abstract

Polycomb repressive complex 2 (PRC2) is a histone methyltransferase critical for maintaining gene silencing during eukaryotic development. In mammals, PRC2 activity is regulated in part by the selective incorporation of one of two paralogs of the catalytic subunit, EZH1 or EZH2. Each of these enzymes has specialized biological functions that may be partially explained by differences in the multivalent interactions they mediate with chromatin. Here, we present two cryo-EM structures of PRC2:EZH1, one as a monomer and a second one as a dimer bound to a nucleosome. When bound to nucleosome substrate, the PRC2:EZH1 dimer undergoes a dramatic conformational change. We demonstrate that mutation of a divergent EZH1/2 loop abrogates the nucleosome-binding and methyltransferase activities of PRC2:EZH1. Finally, we show that PRC2:EZH1 dimers are more effective than monomers at promoting chromatin compaction, and the divergent EZH1/2 loop is essential for this function, thereby tying together the methyltransferase, nucleosome-binding, and chromatin-compaction activities of PRC2:EZH1. We speculate that the conformational flexibility and the ability to dimerize enable PRC2 to act on the varied chromatin substrates it encounters in the cell.

## Abstract (web summary)

Polycomb Repressive Complex 2 (PRC2) is a histone methyltransferase whose silencing activity is regulated in part by the selective incorporation of its catalytic subunits EZH1 or EZH2. Here, the authors capture an EZH1-containing PRC2 dimer on a nucleosome, demonstrating significant conformational changes during the process.

## Introduction

Many mechanisms exist for establishing and maintaining genes in either an “on” or “off” state during early development. Polycomb group (PcG) proteins are a key class of proteins involved in the maintenance of stable silencing of genes^1^. PcG proteins form multisubunit complexes that interact with chromatin to maintain gene repression^2^. Polycomb repressive complex 2 (PRC2) is minimally comprised of four proteins: enhancer of zeste homolog 1/2 (EZH1/2), suppressor of zeste 12 (SUZ12), embryonic ectoderm development (EED), and retinoblastoma-associated protein 46/48 (RBAP46/48)^3,4^. Structurally, PRC2 is organized into an upper catalytic lobe comprised of EZH1/2, EED, and the C terminus of SUZ12, and a lower regulatory lobe containing RBAP46/48 and the N terminus of SUZ12^5^. One of the key biological activities of PRC2 is to methylate histone H3 at lysine 27 (H3K27me), with H3K27me2/3 being most relevant for repression^6,7^. H3K27me3 interacts with PRC2 through an aromatic cage in EED, resulting in the allosteric activation of PRC2 and initiating a positive feedback mechanism that promotes the maintenance of silent PRC2 domains in the genome^8–11^. Interestingly, a mutation to methionine at H3K27 (H3K27M) inhibits PRC2 activity and is the causative aberration in diffuse intrinsic pontine glioma, though the precise mechanism is currently under debate^12–14^. One way that PRC2 activity is regulated is by the selective incorporation of paralogs and/or different accessory factors to form noncanonical complexes^7^. For example, incorporation of either AEBP2 or JARID2 into the core complex increases the nucleosome-binding and methyltransferase activities of PRC2^15–18^. In addition, the catalytic subunit, EZH2, can be substituted by EZH1, the product of a gene-duplication event^19^. PRC2 complexes containing EZH1 (PRC2:EZH1) differ from PRC2:EZH2 in several aspects. For example, EZH2 expression is highest in early mouse tissue and then decreases during development, while EZH1 expression remains constant^20^. Deletion of EZH2 in mice results in embryonic lethality, whereas EZH1 null mice are viable and fertile^21^. However, EZH1 seems to be more important for some adult tissues, such as maintaining adult hematopoietic stem cells through the regulation of multipotency genes^22,23^. These and other differences in the biological roles of PRC2:EZH1 and PRC2:EZH2 are likely linked to differences in functional activities. While PRC2:EZH2 exhibits a higher intrinsic methyltransferase activity for chromatin and is allosterically stimulated by H3K27me3, PRC2:EZH1 has a higher affinity for chromatin and higher chromatin-compaction activity, suggesting that it may be more directly involved in gene silencing^20^. Finally, PRC2:EZH2 occupancy at some genes is co-dependent on EZH1, and EZH1/2 has been shown to interact with each other independent of chromatin, suggesting a possible role for heterodimerization in the regulation of PRC2^17,20,24^. Dimerization may play a more general role in the regulation of PRC2 activity. PRC2 was shown to form dimers in vitro, but the dimerization surfaces and the function of dimers have remained elusive^20,24,25^. A recent crystal structure of a minimal PRC2-dimerization interface demonstrated that PRC2 can dimerize through reciprocal interactions of the SUZ12 N-terminal C2 domain with a surface of RBAP48 in the lower lobe of PRC2^26^. Mutation of three basic amino acids in the C2 domain of SUZ12 disrupted dimerization of the four-component PRC2, reduced CpG island DNA-binding activity, and reduced H3K27 trimethylation at some developmental genes, providing a possible biological role for PRC2 dimerization^26^. Despite these studies, it is not understood molecularly how dimers form in the context of PRC2 containing full-length SUZ12, EED, and EZH1/2 in addition to RBAP48, and how dimers interact with chromatin. In addition, while several high-resolution structures of PRC2:EZH2 have been reported, none explains the functional differences observed for PRC2:EZH1^27–29^. Furthermore, several questions remain regarding the structural heterogeneity of the upper lobe of PRC2 relative to the lower lobe. For example, the four-component PRC2:EZH2 is conformationally flexible, and determining its structure by electron microscopy (EM) required the addition of AEBP2 to stabilize the upper catalytic lobe with respect to the lower regulatory lobe^5^. Lastly, a cryo-EM structure of PRC2:EZH2–AEBP2–JARID2 bound to a dinucleosome did not reveal density for the lower lobe, suggesting that this part of the complex is still highly flexible when PRC2 is bound to nucleosomes, even in the presence of stabilizing factors^30^. These studies suggest that the PRC2 upper and lower lobes exist in alternative conformations relative to each other. However, to date, none of the X-ray or cryo-EM structures have revealed a large structural rearrangement of the two PRC2 lobes.

Here, to understand the differences between PRC2 containing EZH1 or EZH2, we determined the cryo-EM structure of PRC2:EZH1 with two accessory factors, AEBP2 and JARID2. We show that two charged regions in a flexible loop between the MCSS and SANT2L domains of EZH1/2 partially explain differences in functional activities of EZH1 and EZH2. We also determined a structure of PRC2:EZH1 bound to a nucleosome containing the H3K27M “onco-histone”. This structure captures a PRC2:EZH1 dimer on a nucleosome containing H3K27M, with each PRC2:EZH1 in the dimer exhibiting an extensive structural rearrangement compared to the monomeric, nucleosome-free form. This structure of a PRC2 dimer shows not only both the upper catalytic and lower regulatory lobes but also reveals a dramatically different conformation of PRC2. Furthermore, we show that PRC2:EZH1 dimers more effectively promote compaction of nucleosome arrays, suggesting that dimerization helps to coalesce chromatin into silent domains.

## Results

### Cryo-EM structures of free and nucleosome-bound PRC2:EZH1 highlight conformational flexibility

**Fig. 1:**
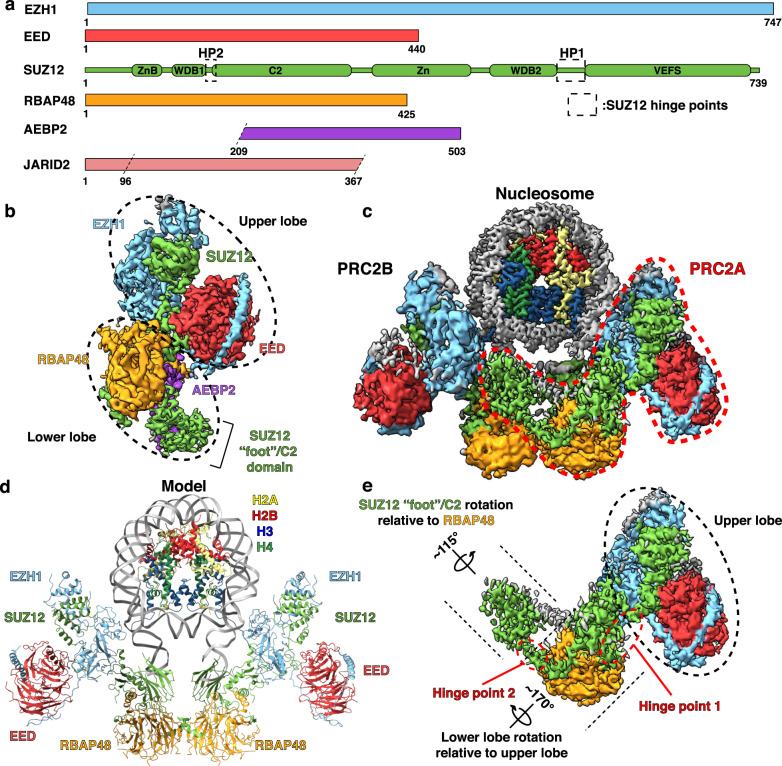
Structures of monomeric and dimeric PRC2:EZH1. **a** Cartoon representation of the proteins used to generate PRC2:EZH1 complexes. Hinge points (HP) 1 (aa 535–561) and 2 (aa 146–155) are regions in SUZ12 that appear to allow the upper and lower lobes to rotate with respect to each other and the C2 domain to rotate with respect to RBAP48. JARID2 amino acids 96–367 were used in the monomeric PRC2 complex, while amino acids 1–367 were used in the PRC2–nucleosome complex. **b** Cryo-EM map of monomeric PRC2:EZH1 showing the upper and lower lobes, and the SUZ12”foot”/C2 domain. Colors are as in panel **a**. **c** Composite cryo-EM map of a PRC2:EZH1 dimer bound to a nucleosome containing the H3K27M and H2AUb modifications. Protein subunits are colored as in panel **a**. One unit of the PRC2 dimer “PRC2A” is outlined with a dashed line. **d** Model of the PRC2:EZH1 dimer bound to a nucleosome containing the H3K27M and H2AUb modifications with subunits labeled. The model was built using the composite map except for nucleosomal DNA, which was modeled based on the 7-Å resolution map. **e** Cryo-EM map of a single PRC2:EZH1 monomer from the structure of the nucleosome-bound PRC2:EZH1 dimer. Compare to map of monomeric PRC2:EZH1 in panel **b**. Note that the lower lobe rotates ~170° relative to the upper lobe, and the C2 domain rotates ~115° relative to RBAP48.

To uncover structural differences that may explain the functional differences between EZH1 and EZH2, we pursued a cryo-EM structure of PRC2:EZH1 for comparison with the known structure of PRC2:EZH2^27–29^. Using a five-component PRC2 containing human EZH1, EED, SUZ12, RBAP48, and AEBP2 (Fig. 1a), we determined a structure at 4.1-Å resolution (Supplementary Fig. 1). The addition of a biologically active fragment of JARID2, containing residues 96–367 (Fig. 1a), allowed us to improve the resolution to 3.9 Å (Fig. 1b and Supplementary Figs. 2, 3a–c)^15,18^. Hereafter, PRC2:EZH1 refers to PRC2 containing EZH1, EED, SUZ12, RBAP48, AEBP2, and JARID2 unless otherwise stated. To build a model of PRC2:EZH1, we docked available X-ray and cryo-EM structures^28,29,31,32^ into our map, made adjustments, and built manually where necessary (Supplementary Fig. 3d, e). Overall, PRC2:EZH1 displays the expected bipartite structure with an upper catalytic lobe containing EZH1, EED, and the C-terminal VEFS domain of SUZ12, and a lower regulatory lobe containing RBAP48, the N terminus of SUZ12, the C terminus of AEBP2, as well as the JARID2 fragment. The two lobes are linked primarily through extensive interactions of SUZ12 with both EZH1 and RBAP48 (Fig. 1b). Aside from a different build of AEBP2 (Supplementary Fig. 3f, g), the overall structure of PRC2:EZH1 is very similar to that of PRC2:EZH2^28^. However, we did note that several loops are disordered in both PRC2:EZH1 and PRC2:EZH2, making us wonder whether any functional differences could be explained by differences contained in these loops (Supplementary Fig. 4a, b). Indeed, sequence alignment of these loops between EZH1 and EZH2 revealed several differences in basic and acidic patches (Supplementary Fig. 4c). We hypothesized that these loops are likely to be involved in chromatin binding and could therefore become ordered when PRC2 binds nucleosomes. To obtain a PRC2:EZH1–nucleosome structure, we first purified a PRC2:EZH1 with a longer JARID2 fragment containing residues 1–367 that includes a ubiquitin-interaction domain^33,34^. We incubated this complex with nucleosomes containing ten base pair (bp) DNA overhangs on either end of a 601 nucleosome-positioning sequence and a histone octamer containing two modifications: histone H3 mutated to methionine at lysine 27 (H3K27M) and histone H2A monoubiquitinated at lysine 119 (H2AUb). H2AUb is deposited through the activity of another polycomb complex, PRC1, and enhances PRC2 activity in part by interactions with JARID2^33,34^. We have previously used ubiquitinated histones to stabilize the binding of chromatin proteins^35^, and under certain conditions H3K27M can greatly enhance PRC2 binding^14^. We reasoned that these nucleosome modifications would help us to capture PRC2:EZH1 bound to a nucleosome. We incubated PRC2:EZH1 with the modified nucleosomes and generated a cryo-EM map at an overall resolution of ~7 Å (Supplementary Fig. 5). Interestingly, our reconstruction revealed a dimer of two PRC2:EZH1 complexes bound to one nucleosome. We used signal subtraction and focused refinement to improve each component in the map individually and were able to refine the nucleosome to an overall resolution of 3.3 Å, one PRC2:EZH1 monomer to 4.1 Å, and the other monomer to 4.8 Å (Fig. 1c and Supplementary Figs. 5c, d, 6a–c). We generated a composite model for the PRC2:EZH1–nucleosome complex by using rigid-body fitting to place individual domains from our model of the PRC2:EZH1 monomer into the map (Fig. 1d). The model shows that the two PRC2:EZH1 complexes bind to opposite sides of the nucleosome and engage with DNA near the locations where the histone H3 N-terminal tails emerge from the nucleosome core particle. We observed clear cryo-EM density consistent with the H3 N-terminal tail interacting with the catalytic site of EZH1 (Supplementary Fig. 6d). We cannot unambiguously assign density for ubiquitin, AEBP2, or JARID2 in our map (see below). The 10-bp DNA ends emerging from the nucleosome are in proximity to a SUZ12 α-helix (aa 80–107) (Supplementary Fig. 6e). In our lower-resolution maps, we can see density for the DNA that becomes fragmented upon focused refinements, suggesting that this DNA is flexible. The expected DNA trajectory places nucleosome linker DNA in a location that allows for interaction with SUZ12/RBAP48. The N terminus of SUZ12 is required for genomic localization of PRC2 at CpG islands, possibly through direct or indirect mechanisms^36^. Further experiments are required to determine whether this SUZ12 α-helix plays a direct role in PRC2 localization.

A comparison with the PRC2:EZH1 monomer reveals that some domains in the PRC2:EZH1 dimer have undergone a large conformational rearrangement (Supplementary Movie 1). Relative to the upper lobe of PRC2:EZH1, the lower lobe rotates ~170°, pivoting through an apparent hinge point (aa 535–561) in SUZ12 (Fig. 1a, e). In addition, the C2 domain of SUZ12, which normally interacts with a surface of RBAP48 within a PRC2:EZH1 monomer, rotates ~115° through an additional apparent hinge point (aa 146–155) to interact with the surface of RBAP48 of the second PRC2:EZH1 complex, resulting in a domain swap (Fig. 1a, e and below). Overall, the modified nucleosome allowed us to capture a PRC2:EZH1 dimer on a single nucleosome, revealing remarkable flexibility between the upper and lower lobes, which appears to be enabled by SUZ12 segments acting as hinge points (Fig. 1a).

### A disordered loop near the SANT2L domain of EZH1 contributes to substrate binding and catalytic activity of PRC2

**Fig. 2:**
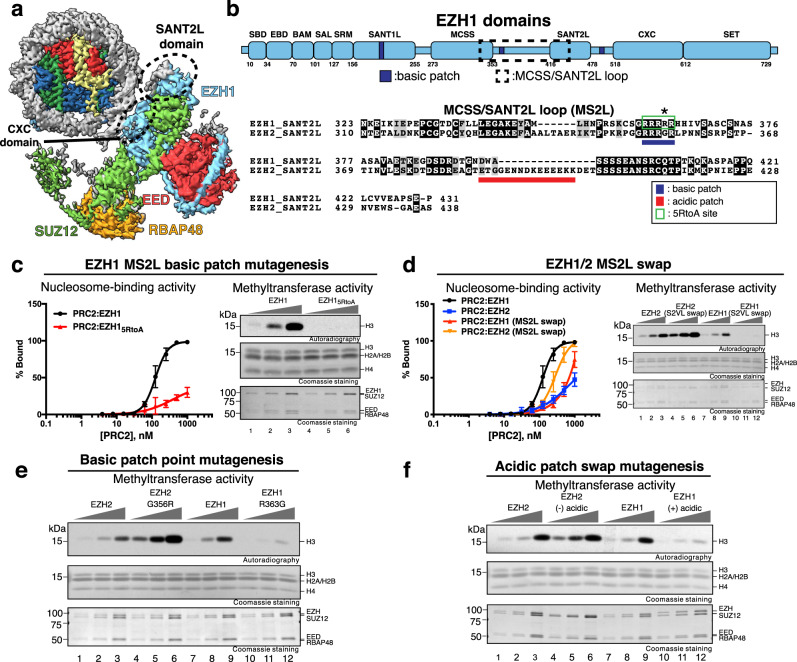
The MCSS/SANT2L loop is important for PRC2 activity. **a** Composite map of one PRC2:EZH1 bound to nucleosome with regions that likely interact with DNA indicated by dashed circles. **b** Upper panel: Cartoon representation of EZH1 showing basic patches. Lower panel: Sequence alignment of EZH1 and EZH2 MCSS/SANT2L loops showing the basic patch (green box) and the acidic patch (red underline). **c** Nucleosome-binding and methyltransferase activities of PRC2:EZH1 containing five arginine-to-alanine substitutions (see green box in panel **b**). Experiments were done with core PRC2 containing SUZ12, EED, RBAP48, and wild-type or mutant versions of EZH1/2 as indicated. For graphs indicating nucleosome binding, each data point represents the average of three independent mobility-shift experiments and is shown as mean ± standard deviation. Methyltransferase assays measured the incorporation of ^3^H-labeled S-adenosylmethionine into histone H3 at lysine 27. Assays were done in triplicate using 300 nM of nucleosomes and titration of 5–60 nM PRC2. **d** Experiments done as in panel **c**, except that regions aligned in panel **b** were “swapped” between EZH1 and EZH2. **e** Methyltransferase assay using reciprocal point mutation swaps between EZH1 and EZH2 (EZH1-R363G vs EZH2-G356R) (asterisk in panel **b**). The experiment was done as in panel **c**. **f** Methyltransferase activity of PRC2 complexes with the acidic patch (see panel **b**) deleted from EZH2 or inserted into EZH1 (residues 387–401 from EZH2 deleted or inserted into EZH1 between residues 394 and 395). The experiment was done as in panel **c**.

In our model of the nucleosome-bound PRC2:EZH1 dimer, the primary contact to nucleosomal DNA occurs through the CXC motif of EZH1 (Fig. 2a). This primary contact and EZH1 orientation is similar to one of the nucleosome interactions observed in a cryo-EM structure of monomeric PRC2:EZH2 bound to a dinucleosome^30^. Interestingly, this places a divergent EZH1/2 loop that we call the MCSS/SANT2L loop (MS2L) near nucleosomal DNA (Fig. 2a). This loop is located within a part of EZH1 that has previously been shown to contribute to nucleosome binding^17^. Since this loop contains a patch of basic amino acids, we wondered if it plays a role in nucleosome interactions and/or the methyltransferase activity of PRC2:EZH1 (Fig. 2b). To test this possibility, we generated an EZH1 version in which we mutated the five consecutive arginines in the basic patch (green box in Fig. 2b) to alanines (5RtoA mutation). Previously, we have shown that the functional differences between EZH1/2 are highest in the four-component PRC2 complexes^18^. Since we wanted to compare differences conferred by EZH1/2, we incorporated the mutant protein into the four-component PRC2 complex and tested its nucleosome-binding and methyltransferase activities. PRC2:EZH1_5RtoA_ showed severely decreased nucleosome-binding activity and no detectable methyltransferase activity (Fig. 2c). To control for the possibility of nonspecific charge effects on the methyltransferase and nucleosome-binding activities, we generated a mutant version of EZH1, in which we mutated five random arginines spread throughout the protein to alanine (R31,64,100,321,443A). We used this EZH1 variant to form a PRC2 and tested its nucleosome-binding and methyltransferase activities. This complex showed slightly reduced nucleosome-binding activity and no change in methyltransferase activity (Supplementary Fig. 7a, b). Therefore, we conclude that the loss of activity in the PRC2:EZH1_5RtoA_ complex is not due to an overall reduction in charge. Since the MS2L of EZH1 differs in several places from that of EZH2 and is adjacent to nucleosomal DNA, we wondered whether any of these differences could explain the stronger chromatin-binding and compaction activities of PRC2:EZH1^20^. Notably, the MS2L in EZH2 has an arginine-to-glycine substitution in the basic patch and contains an acidic insertion (Fig. 2b). We swapped the entire MS2L between EZH1 and EZH2, generated complexes, and tested their nucleosome-binding and methyltransferase activities. As seen in the left panel of Fig. 2d, this swapping reversed the intrinsic nucleosome-binding activities of the complexes, suggesting that the MS2L of EZH1 is partially responsible for the higher nucleosome-binding activity of EZH1. Most importantly, the stronger nucleosome-binding activity conferred by the EZH1 MS2L resulted in enhanced methyltransferase activity of the complexes, demonstrating that the nucleosome-binding activity is directly involved in enhancing catalysis. Therefore, high nucleosome-binding activity directly correlates with increased methylation activity, and these functions are linked through MS2L. Finally, we asked whether a single-residue substitution, (asterisk in Fig. 2b) or a domain swap of the acidic region of EZH2 (see Fig. 2 legend) impacted methyltransferase activity (Fig. 2e, f). Each of these mutations did affect the complexes’ methyltransferase activities, with the more basic versions of the mutants being more potent. Thus, these data suggest that the MS2L loop underlies part of the functional differences between EZH1 and EZH2, and that these differences are explained by divergent patches of charged residues. Furthermore, these results are consistent with previously published work showing the importance of the SANT2L region for conferring differences in nucleosome-binding and methyltransferase activities to PRC2 containing EZH1/2^17^. We note, however, that these experiments do not determine whether dimerization impacts the functions of the MS2L loop.

### Structure and function of the dimeric form of PRC2

**Fig. 3:**
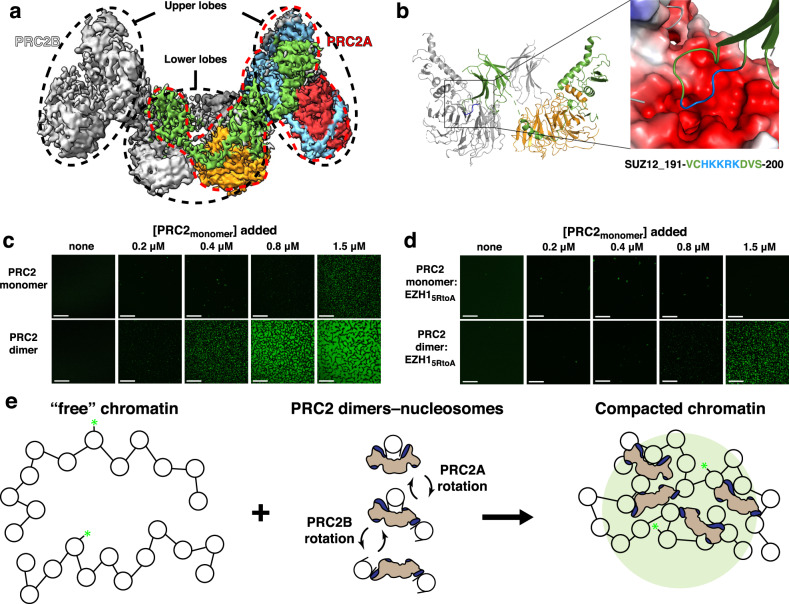
PRC2 dimers promote chromatin compaction. **a** Cryo-EM map showing the PRC2:EZH1 dimer. Subunits are colored as in Fig. 1. One PRC2 monomer is highlighted in color and by a red dashed line. **b**, left panel: Isolated view of the dimeric interface of PRC2:EZH1 showing that the C2 domain of SUZ12 (green) from one PRC2:EZH1 interacts with RBAP48 from the other PRC2:EZH1 (gray). Right panel: Zoomed-in view of the basic loop (blue) in the C2 domain interacting with the acidic surface of RBAP48. Electrostatics were calculated using APBS in PyMol. The sequence of the basic residues in the C2 domain is shown and colored blue. **c** Confocal micrographs of serial twofold dilutions of PRC2:EZH1 mixed with nucleosome arrays showing compaction of chromatin into droplets. Scale bar: 100 μm. The final concentration of PRC2 in the reactions is indicated. Molarity is based on the expected size of PRC2:EZH1 monomers. All samples contain 3 μM total nucleosomes, assembled into arrays with 12 nuclesomes spaced 40 bp apart. Experiments were repeated at least three times with independently purified complexes and were consistent with each other. **d** Chromatin compaction as promoted by PRC2:EZH1_5RtoA_ mutant complexes. Concentrations and scale are as in **c**. Experiments were repeated twice with independently purified complexes that were consistent with each other. **e** Model of chromatin compaction promoted by PRC2 dimers. Chromatin is compacted into a phase-separated state through multivalent interactions with basic patches of PRC2:EZH1 dimers, leading to the formation of liquid–liquid phase-separated droplets.

In our structure of nucleosome-bound PRC2:EZH1, we observed PRC2:EZH1 homodimer formation through interactions of the lower lobes composed of SUZ12 and the histone-binding protein RBAP48 (Fig. 3a). The dimer interface occurs through a domain “swap”, in which the C2 domain of SUZ12 from one PRC2:EZH1 complex interacts with RBAP48 from the other. This interaction appears to be mediated by a loop of positively charged residues on SUZ12 (H193-K197) binding to a negatively charged surface on RBAP48 (Fig. 3b). We also observe this same loop interacting with the acidic surface of RBAP48 in our structure of monomeric PRC2:EZH1 (Supplementary Fig. 8a, b). This domain swap was also seen in a recent crystal structure of the PRC2 lower lobe^26^. While the basic loop/RBAP48 interactions and overall architecture seen in this structure are generally the same, we note that the SUZ12 C2 domain and RBAP48 adopt different conformations in the crystal structure, highlighting the flexibility of the dimerization interface within PRC2 (Supplementary Fig. 8c). We do not note anything that would prevent four-component PRC2 complexes from forming hetero- or homo-dimers of PRC2:EZH1 and PRC2:EZH2 in cells containing both complexes.

We wondered what function dimerization could play in the regulation of PRC2 activity. First, we separated monomeric and dimeric PRC2:EZH1 using size-exclusion chromatography and confirmed the PRC2:EZH1 dimer by using size-exclusion chromatography coupled to multiangle light scattering and negative-stain EM (Supplementary Fig. 9a–c). Interestingly, the purified monomers and dimers did not freely interconvert in solution over a 24-h incubation at 4 °C, suggesting that in isolation the oligomeric state is stable (Supplementary Fig. 9d). In order to verify the role of the SUZ12 basic patch in stabilizing the PRC2:EZH1 dimer, we expressed and purified a version of the complex with SUZ12 amino acids 193–197 mutated to alanine (SUZ12_193-197A_). As expected, this cluster of mutations completely prevented the dimerization of PRC2:EZH1 (Supplementary Fig. 10a). Next, we tested the monomeric, dimeric, and mutant fractions of PRC2:EZH1 in our nucleosome-binding and methyltransferase assays. We could not detect any appreciable difference in apparent nucleosome-binding activity, and only observed modest differences in methyltransferase activity, suggesting that dimerization does not play a primary role in these activities of PRC2:EZH1 under the conditions tested (Supplementary Fig. 10b, c). The lack of differences in nucleosome-binding and methyltransferase activities observed in our SUZ12_193-197A_-containing complex further confirms that the effect of our PRC2:EZH1_5RtoA_ mutation on these activities is specific and not due to a change in the overall charge of PRC2:EZH1 (Fig. 2c).

Since dimerization of PRC2:EZH1 did not impact the methyltransferase and nucleosome-binding activities, we reasoned that dimerization might affect its chromatin-compaction activity. To test this idea, we employed an in vitro assay using fluorescently labeled nucleosome arrays that measure the propensity of chromatin to coalesce^37^ (Fig. 3c). When we added increasing amounts of PRC2:EZH1 monomer to fluorescein-labeled nucleosome arrays, we first detected the formation of droplets at a PRC2:EZH1 monomer concentration of 0.8–1.5 μM. In contrast, when we repeated the experiment with PRC2:EZH1 dimers, we observed the first droplets at a PRC2:EZH1 (monomer) concentration of 0.2–0.4 μM, an approximately fourfold increase in activity (note that molarity is based on the size of monomeric PRC2:EZH1). This finding is consistent with PRC2:EZH1 dimerization functioning to promote multivalent interactions within heterochromatin leading to compaction. As expected, PRC2:EZH1 containing SUZ12_193-197A_ was ineffective at chromatin compaction (Supplementary Fig. 10d).

We wondered if the MS2L basic patch contributed to the activity of PRC2:EZH1 dimers. Since the 5RtoA mutation severely inhibited the nucleosome-binding and methyltransferase activities of the PRC2:EZH1 core complex (Fig. 2), we decided to test if this mutation also impacts nucleosome-array compaction. When we tested the mutant complex, we observed no promotion of nucleosome-array compaction by the monomeric complex at the highest PRC2:EZH1 concentration tested, and we saw a fourfold reduction in the activity of the dimeric complex, consistent with the basic patch of PRC2 being involved in promoting chromatin compaction (Fig. 3d). These results demonstrate that PRC2:EZH1 dimers are more effective than monomers in chromatin compaction, and show that, in addition to the methyltransferase and nucleosome-binding activities, the MS2L loop is required for full compaction activity in our assay (Fig. 3e).

Finally, we compared the ability of PRC2:EZH1 and PRC2:EZH2 dimers to compact chromatin in this assay. Previously, PRC2-containing EZH1 has been shown to compact chromatin more effectively than PRC2 containing EZH2^20^. PRC2:EZH2 purified on size-exclusion chromatography consistently showed a larger population of dimers than PRC2:EZH1, suggesting that EZH2 has a stabilizing effect on the dimeric form of PRC2 (Supplementary Fig. 11a). However, when we compared the complexes in our assay, PRC2:EZH1 was more effective in compacting chromatin (Supplementary Fig. 11b). Taken together, these results suggest that both dimerization and the nucleosome-binding activity are required for efficient chromatin compaction.

## Discussion

We have determined structures of monomeric PRC2:EZH1 and a homodimer of PRC2:EZH1 bound to a nucleosome. Together, these structures show that PRC2 can adopt different conformations and highlight the flexibility of the upper and lower lobes with respect to each other.

We used two histone modifications to attempt to stabilize PRC2:EZH1 on nucleosomes, H2AUb and H3K27M. There are at least three possible reasons why we do not observe density for H2AUb. (1) Ub may interact with PRC2:EZH1 in many different orientations so that it becomes averaged out during image processing. (2) Since Ub is linked to the end of a histone tail, it may not directly interact with PRC2:EZH1 in our dimeric complex and thus be mobile, again causing it to be averaged out during image processing. (3) Dimerization of PRC2:EZH1 on a nucleosome leads to the eviction of AEBP2 and JARID2, which in turn prevents the ubiquitin-interaction domain of JARID2 from interacting with H2AUb, preventing the stabilization of each other. While AEBP2 and JARID2 have a stabilizing effect on monomeric PRC2, AEBP2 seems to inhibit the formation of PRC2 dimers^26^. Consistent with this notion, we were unable to unambiguously assign any density to either cofactor in our PRC2:EZH1 dimer–nucleosome complex, suggesting that they may be evicted during the dimerization process. In addition, we cannot rule out the possibility that this conformation of PRC2:EZH1 is exclusive to mononucleosomes containing H2AUb and H3K27M. Further studies of monomeric and dimeric PRC2 in vitro and in vivo will be necessary to determine how these forms interact with modified and unmodified nucleosome arrays. However, the nucleosome interaction does not appear to be necessary for the flexibility in dimeric PRC2:EZH1, as we observed multiple species in our negative-stain EM averages of the dimer in solution (Supplementary Fig. 9c) that are consistent with the reorganization of the upper and lower lobes we describe in Fig. 1e.

We find that PRC2 has two primary modes of structural flexibility that affect its function: (1) EZH1/2 contain flexible loops with patches of charged amino acids that create differences in chromatin interactions and methyltransferase activity, and (2) the upper and lower lobes of PRC2:EZH1 are connected in a way that allows substantial structural rearrangements, which we speculate is important for forming multivalent interactions with diverse chromatin substrates that leads to chromatin compaction.

PRC2 contains many flexible loops. We found a discrete stretch of basic amino acids in a flexible loop adjacent to the SANT2L domain of EZH1/2 that contributes to PRC2 functional activities. A common feature in eukaryotic transcription regulatory proteins are flexible regions that contain low sequence complexity and are intrinsically disordered^38^. While intrinsically disordered regions often contain gene activation or repression activities, they are not fully understood. In PRC1, basic intrinsically disordered regions within the CBX2 protein promote nucleosome compaction in vitro and gene silencing in vivo and have been implicated in promoting phase separation^39–42^. We speculate that basic or acidic patches in chromatin-interacting proteins are a common regulatory feature that promotes multivalent interactions between charged chromatin surfaces, such as basic histone tails, DNA phosphates, or the acidic patch on the surface of the nucleosomes, and helps drive compaction of chromatin domains.

Chromatin is very heterogeneous with regard to nucleosome positioning and overall architecture, owing to the dynamic needs of regulating transcription of thousands of genes in higher eukaryotes. We found a large structural change in PRC2:EZH1 when it dimerized on a single nucleosome. Since the major lobe movement consists of a rotation around apparent SUZ12 hinge point 1, we speculate that PRC2-containing EZH2 can also adopt different conformations. In addition, since the primary dimerization interface is located on subunits that are shared between PRC2:EZH1 and PRC2:EZH2, this surface would allow the formation of both homo- and heterodimers of PRC2:EZH1 and PRC2:EZH2^20,24,25^. This could explain the observation that EZH1 is required for EZH2 localization at some genomic targets, and would provide an additional layer of gene regulation^17^.

We found that PRC2:EZH1 dimers are approximately fourfold more effective than monomers at compacting chromatin. One of the predicted functions of PRC2:EZH1 is to maintain heterochromatin in a compact form, similar to the role of linker histone H1, CBX2-containing PRC1, and heterochromatin protein HP1α. All of these proteins/complexes have been suggested or shown to be involved in chromatin compaction^37,40–44^. Like PRC2, chromatin compaction by HP1α depends on both dimerization and flexible patches of basic amino acids, suggesting that dimerization creates molecules with multiple regions poised to form electrostatic interactions with chromatin. Since multivalent interaction domains are frequently found in proteins involved in establishing and maintaining silent domains, we speculate that dimerization may play a role in the spreading of PcG domains along chromatin fibers to maintain gene repression. Multivalent interactions and dimerization are a general feature of gene-silencing complexes^45^. Importantly, having multiple chromatin-binding modules that are flexibly tethered within one complex would be expected to improve multivalent interactions with diverse nucleosome architectures (Fig. 3e). A recent manuscript with a crystal structure of the dimerization interface of PRC2 elegantly demonstrated that mutation of basic residues in the SUZ12 C2 loop results in loss of dimerization and reduction in PRC2 occupancy at some genes, suggesting that dimerization may play a role in recruitment or stabilization at some genomic targets^26^. Further experiments will be required to determine the role of dimerization at the organismal level.

## Methods

### Protein expression and purification

#### PRC2 core complex

Codon-optimized versions of human Strep-EZH1, Flag-SUZ12, His-EED, and His-RBAP48 were cloned into the pBIG1a baculovirus expression plasmid, and the sequence was verified^46^. Bacmids were generated in DH10Bac cells and used to transfect Sf9 cells using Bac-to-Bac protocols (Life Technologies). Baculovirus was used to infect Sf9 or Hi5 cells for expression and harvested 60 h post infection. Cells were lysed in Buffer A (20 mM Tris-HCl, pH 7.5, 300 mM NaCl, 10% v/v glycerol, 1 mM DTT) plus Roche cOmplete protease inhibitors using an Emulsiflex C3 homogenizer (Avestin). Lysates were clarified by centrifugation at 40,000 × *g* for 20 min at 4 °C, then frozen or further processed. Lysates were bound to Streptavidin beads (Strep-Tactin Macroprep, IBA) for 2 h, then washed extensively in Buffer A without protease inhibitors. Bound PRC2 was cleaved overnight with HRV3C and thrombin in Buffer A at 4 °C. Eluted protein was dialyzed into Q-low buffer (20 mM Tris-HCl, pH 7.5, 150 mM NaCl, 1 mM DTT), bound to a HiTrap Q FF column (GE Healthcare), and eluted with a linear gradient of Q-high buffer (20 mM Tris-HCl, pH 7.5, 1 M NaCl, 1 mM DTT). Fractions containing PRC2 were pooled, concentrated, and loaded onto a Superose 6 Increase column (GE Healthcare) equilibrated with Buffer A. Fractions containing PRC2 were pooled, concentrated, and flash-frozen.

#### EZH1 and SUZ12 variants

Mutant versions of EZH1 and SUZ12 were cloned into the pBIG1a expression vector containing the other core subunits of PRC2 and sequence verified. Expressions and purifications were followed according to the protocol for wild-type PRC2.

#### AEBP2

The short isoform of AEBP2 (aa 209–503; UniProt: Q6ZN18) was cloned into pGEX6P1 plasmid and used to transform *E. coli* BL21 cells. Cultures were grown until OD600 reached 0.6, then induced with 0.4 mM isopropyl β-D-1-thiogalactopyranoside (IPTG). Cells were grown for 2 h at 37 °C, then harvested by centrifugation at 3500 × *g* for 20 min. Cells were resuspended in Buffer A plus protease inhibitors, lysed using an Emulsiflex C3, and clarified by centrifugation at 40,000 × *g* for 20 min. Lysates were bound to Protino glutathione agarose 4B resin (Macherey-Nagel) for 2 h at 4 °C, then extensively washed with Buffer A. Beads were resuspended in 10 ml of HiTrap S buffer (20 mM HEPES, pH 7.5, 300 mM NaCl, 1 mM DTT) and cleaved overnight with HRV3C protease at 4 °C. Eluted protein was bound to a HiTrap S column (GE Healthcare) and eluted with a linear gradient of HiTrap S buffer containing 1 M NaCl. Fractions containing AEBP2 were pooled, concentrated, and flash-frozen.

#### JARID2

The JARID2_1-367_ and JARID2_96-367_ fragments were cloned into pGEX6P1 plasmid using standard cloning techniques. Plasmids were transformed into *E. coli* BL21 RIL cells and overnight cultures were grown. Large-scale cultures were inoculated and grown to OD_600_ of 0.4–0.6 at 37 °C, then shifted to 18 °C prior to induction with 250 μM IPTG. Cultures were grown for ~18 h and harvested by centrifugation at 3500 × *g* for 10 min. Lysates were processed the same as AEBP2. After binding to Protino glutathione agarose 4B resin, protein was eluted using GST-elution buffer (40 mM reduced glutathione, 50 mM Tris-HCl, pH 8.0, 1 mM MgCl_2_, 500 mM NaCl, 20% v/v glycerol, and 5 mM DTT). Fractions were combined and dialyzed overnight at 4 °C into HIC buffer A (1 M ammonium sulfate, 10 mM Tris-HCl, pH 7.5, 1 mM DTT) and loaded onto a HiTrap butyl sepharose column. Protein was eluted with a linear gradient of HIC buffer B (10 mM Tris-HCl, pH 7.5, 1 mM DTT). Fractions containing JARID2 were simultaneously cleaved with HRV3C and dialyzed overnight at 4 °C into Mono Q buffer A (20 mM HEPES, pH 7.6, 50 mM KCl, 5% v/v glycerol, 1 mM DTT). Free GST and uncut protein were removed by passing the sample over glutathione sepharose resin. The flow-through was loaded onto a Mono Q column and eluted with a linear gradient of Mono Q buffer containing 1 M KCl. Fractions containing JARID2 were pooled and flash-frozen.

#### PRC2 plus AEBP2 and JARID2

To purify core PRC2 with accessory factors, 1.5 molar excess of either AEBP2 alone or AEBP2 and JARID2 (either aa 1–367 or 96–367) were mixed with core PRC2 and incubated on ice for 30 min, followed by purification over a Superose 6 Increase column equilibrated with Buffer A. Fractions containing monomeric or dimeric versions of the complexes were pooled and flash-frozen.

#### Histone proteins

Individual *Xenopus laevis* wild-type and mutant histones were expressed and purified based on Luger et al.^47^. Individual histone genes in pET3a vectors were transformed into *E. coli* BL21 DE3 pLysS. Fresh colonies were used to inoculate 5-mL cultures and were grown overnight at 37 °C. The next day 3-L cultures were inoculated from the overnight cultures and grown until OD_600_ of 0.4 at 37 °C. Cultures were induced by adding IPTG to 400 μM and shaken at 37 °C for 2 h. Cells were harvested by centrifugation at 3500 × *g* for 10 min and resuspended in inclusion body wash buffer (50 mM Tris-HCl, pH 7.5, 100 mM NaCl, 1 mM benzamidine, 1 mM β-mercaptoethanol), and flash-frozen. Cell pellets were thawed and lysed with a Tissumizer (Tekmar). Lysed cells were centrifuged 15,000 × *g* for 20 min at 4 °C. The pellets were resuspended with wash buffer plus 1% Triton X-100 and then centrifuged again as before. The Triton X-100 wash was repeated twice more, and then the pellets were washed twice with wash buffer without Triton X-100. After the final wash, 1 mL of DMSO was added to the pelleted inclusion bodies and they were incubated for 30 min. In all, 20 mL of unfolding buffer was added to each pellet (7 M guanidine hydrochloride, 20 mM sodium acetate, pH 5.2, 10 mM DTT) and gently stirred for 1 hr then centrifuged 25,000 × *g* for 10 min. The resulting supernatants were purified on a 2-L column packed with Superdex 200 and equilibrated with SAUDE200 (7 M Urea, 20 mM Sodium Acetate, pH 5.2, 200 mM NaCl, 1 mM EDTA, 5 mM β-mercaptoethanol). The fractions containing histones were pooled and loaded onto a 500-mL SP Sepharose column equilibrated into SAUDE200 and eluted with a linear gradient of SAUDE1000 (7 M urea, 20 mM sodium acetate, pH 5.2, 1000 mM NaCl, 1 mM EDTA, 5 mM β-mercaptoethanol). Fractions containing pure histones were dialyzed extensively into water containing 1 mM β-mercaptoethanol and lyophilized until further use. Ubiquitinated H2A was prepared as described for histone H2B^35^. Histone H2A K119C was expressed and purified according to the protocol for the wild-type histones. To generate ubiquitin, His-tagged ubiquitin G76C in pET was transformed into SoluBL21, and cultures were grown to an OD_600_ of 0.4 at 37 °C and induced with 400 μM IPTG. Cultures were grown for an additional 4 h. Bacterial pellets were resuspended in ubiquitin lysis buffer (50 mM Tris-HCl, pH 8.0, 300 mM NaCl, 10 mM imidazole, 5 mM β-mercaptoethanol, plus Roche cOmplete protease inhibitors) and lysed using an Emulsiflex C3 homogenizer. The lysate was bound to Ni-NTA agarose (QIAGEN) and eluted with lysis buffer plus 300 mM imidazole. Fractions containing ubiquitin were dialyzed into 20 mM Tris-HCl, pH 8.0, 50 mM NaCl, 0.2 mM EDTA, 10 mM β-mercaptoethanol, and then loaded onto a HiTrap Q HP (GE Healthcare) column and eluted with a linear gradient of loading buffer supplemented with 1 M NaCl. Fractions containing ubiquitin were extensively dialyzed into deionized water supplemented with 1 mM acetic acid followed by lyophilization. To prepare cross-linked H2AUb, lyophilized pellets of His-tagged ubiquitin G76C and H2A K119C were dissolved in resuspension buffer (10 mM acetic acid, 7 M urea) to 10 mg/ml. Proteins were mixed to 2:1 molar ratio of Ub to H2A. Sodium tetraborate and TCEP were added to final concentrations of 50 mM and 5 mM, incubated for 30 min on ice. Next, 0.1 M 1,3, dichloroacetone (Sigma) in dimethylformamide (Sigma) was added to 0.5 molar equivalents of sulfhydryl groups and incubated on ice for 1 h. Reactions were quenched by adding β-mercaptoethanol to 5 mM. Reactions were purified over Ni-NTA agarose (QIAGEN), lyophilized extensively against deionized water supplemented with 1 mM β-mercaptoethanol, and stored at −80 °C until used. To generate histone octamers, lyophilized aliquots of the desired individual histones were resuspended in unfolding buffer (7 M guanidine hydrochloride, 20 mM sodium acetate, pH 5.2, 10 mM DTT) for 30 min, then mixed in equimolar ratios. The mixture of histones was dialyzed extensively into refolding buffer (2 M NaCl, 10 mM Tris-HCl, pH 7.5, 1 mM EDTA, 5 mM β-mercaptoethanol). The refolded histone octamers were purified on a Superdex 200 column equilibrated into refolding buffer. The fractions containing octamers were pooled, concentrated, and stored at 4 °C until use.

### Nucleosomal DNA generation

Plasmids containing 601 DNA fragments for assemblies were prepared using large-scale alkaline lysis^48^. Large-scale cultures (12 L) of *E. coli* containing plasmid DNA were grown overnight at 37 °C. Cells were harvested by centrifugation at 3500 × *g* for 10 min, then resuspended with 80 mL of P1 (10 mM EDTA, pH 8) per liter of culture and combined. In total, 160 mL of P2 (0.2 M NaOH, 1% sodium dodecyl sulfate) per liter of culture was added by shaking vigorously and then incubated on ice for 20 min. In all, 160 mL of P3 (4 M potassium acetate, 2 M acetic acid) per liter of culture was added and mixed by gently inverting followed by incubation on ice for 20 min. Lysates were centrifuged at 3500 × *g* for 20 min, and then the supernatants were filtered through miracloth. In all, 0.5 volumes of isopropyl alcohol were added, and the mixture was stirred for 60 min at 4 °C. The precipitated plasmids were centrifuged 3500 × *g* for 30 min, air dried, and resuspended in 50 mL TE buffer. In total, 0.5 mg of RNAse A (Thermo) was added and incubated at 37 °C overnight. Solid potassium chloride was added to the plasmid to adjust the final concentration to 2 M. The mixture was purified over a 500-mL Sepharose 6 column equilibrated in plasmid buffer (50 mM Tris-HCl, pH 7.5, 2 M KCl, 1 mM EDTA). Fractions containing plasmid were pooled and precipitated with isopropyl alcohol, centrifuged 3500 × *g* for 30 min, dried, and resuspended in TE until use. Plasmids were digested with EcoRV to liberate the fragment containing nucleosome-positioning sequences and purified using fractional PEG precipitation. Overnight digestions were mixed with 5 M NaCl to obtain 0.5 M NaCl. A solution of 40% PEG 6000 in 0.5 M NaCl was added to 4.5%, incubated on ice for 1 hr, and centrifuged 25,000 × *g* for 20 min. Supernatants and pellets were run on an agarose gel to monitor precipitation. Additional PEG solution was added in 0.5% increments, and the process was repeated until the desired fragment of DNA was separated from the plasmid backbone. The purified fragments were precipitated with 2.5 volumes of ethanol overnight at −20 °C. DNA was resuspended in TE to a concentration of 2 mg/ml and filtered until use.

### Nucleosome assemblies

Nucleosomes were assembled using gradient dialysis, as described elsewhere^49^. Briefly, purified DNA and histone octamers were assembled in a high salt buffer (2 M KCl, 10 mM Tris-HCl, 7.5, 1 mM EDTA, 1 mM DTT) and dialyzed into low-salt buffer (0.25 M KCl, 10 mM Tris-HCl, 7.5, 1 mM EDTA, 1 mM DTT) via a peristaltic pump over 16 h. After addition, the assemblies were dialyzed for 4 h into a low-salt buffer and then overnight into TE (10 mM Tris-HCl, 7.5, 1 mM EDTA, 1 mM DTT). Nucleosome arrays were stored until used. Mononucleosomes were further purified over a ResourceQ column (GE Healthcare) equilibrated with TE and eluted with TE plus 1 M NaCl. Fractions containing nucleosomes were dialyzed into TE and stored at 4 °C until used. Optimized DNA/octamer ratios were determined empirically using small-scale reactions, and reactions were then scaled up. Nucleosome assemblies were verified by running samples on native PAGE gels.

### Electrophoretic mobility-shift assay (EMSA)

Proteins and nucleosome samples were dialyzed into EMSA binding buffer (EBB) (10 mM HEPES, pH 7.9, 50 mM KCl, 5% v/v glycerol, 5 mM DTT). Serial dilutions of PRC2 proteins were made in EBB, and then an equal volume of 25 nM nucleosomes in EBB plus 0.5 mg/ml BSA was added to each PRC2 dilution. Reactions were incubated at room temperature for 30 min, loaded onto 3.5% native polyacrylamide gels, and run at 120 V for 70 min using 0.3× Tris Borate EDTA (TBE) running buffer (30 mM Tris base, 30 mM boric acid, 0.6 mM EDTA). Gels were stained with SYBR gold (Thermo Fisher) for 15 min and scanned with a Typhoon Imager (GE Healthcare). Scans of the gels are included in the accompanying Source Data File. DNA bands were quantified using ImageJ 2^50^ and graphed using Prism 8 (GraphPad) with data from three independent replicates.

### Methyltransferase assays

Histone methyltransferase (HMT) assays were performed in a total volume of 15 μl containing HMT buffer (50 mM Tris-HCl, pH 8.5, 5 mM MgCl_2_, and 4 mM DTT) with 500 nM of ^3^H-labeled S-adenosylmethionine (PerkinElmer), 300 nM of nucleosomes, and recombinant human PRC2 at the indicated concentrations. Reaction mixtures were incubated for 60 min at 30 °C and stopped by adding 4 μl of STOP buffer (0.2 M Tris-HCl, pH 6.8, 20% v/v glycerol, 10% m/v SDS, 10 mM β-mercaptoethanol, and 0.05% bromophenol blue). A titration of PRC2 (from 5 to 60 nM) was performed under these conditions to establish that the HMT reactions were within the linear range. After the addition of STOP buffer, samples were incubated for 5 min at 95 °C and separated on SDS-PAGE gels. The gels were subjected to Coomassie blue staining for protein visualization and subsequently transferred to 0.45-μm PVDF membranes (Millipore) using standard Western blotting techniques and exposed to autoradiography film (Denville Scientific). All reactions were done in triplicate. Uncropped scans of the gels are included in the accompanying Source Data File. MTase-Glo assays (Promega) were performed according to the manufacturer’s recommendations. Briefly, reactions were assembled in a total volume of 8 μl in MTase-Glo buffer (50 mM Tris-HCl, pH 8.0, 1 mM MgCl_2_, 50 mM NaCl, 0.1 mg/ml BSA, 4 mM DTT) with 10 μM SAM, 1 μM nucleosomes, and recombinant human PRC2 at the indicated concentrations. Reactions were incubated for 20 min at 25 °C and stopped by adding 2 μl of 0.5% trifluoroacetic acid. 2 μl of 5× MTase-Glo reagent was added, and reactions were incubated for 30 min at 25 °C. In total, 10 μl of detection reagent was added and reactions were incubated for 30 min at 25 °C, followed by measuring luminescence in an EnSpire plate reader (PerkinElmer). Reactions were done in triplicate and graphed using Prism 8.

### Cryo-EM sample preparation and data collection

PRC2:EZH1 was cross-linked using the GraFix protocol^51^. Briefly, protein complexes were dialyzed into Buffer GA (20 mM HEPES, pH 7.9, 50 mM KCl, 5% v/v glycerol, 1 mM DTT). A 10–40% glycerol gradient was created in 4 ml of Buffer GB (20 mM HEPES, pH 7.9, 50 mM KCl, 10% v/v glycerol, 1 mM DTT) and Buffer GC (20 mM HEPES, pH 7.9, 50 mM KCl, 40% v/v glycerol, 0.1% v/v glutaraldehyde, 1 mM DTT). PRC2:EZH1 was layered onto the top of the gradient and centrifuged in a SW 60Ti rotor for 14 h at 100,000 × *g*. Fractions were analyzed by SDS-PAGE, and relevant fractions were pooled, concentrated, and dialyzed into freezing buffer (20 mM HEPES, pH 7.9, 50 mM KCl, 1 mM MgCl_2_, 1 mM DTT). To generate PRC2:EZH1 complexes with nucleosomes, PRC2:EZH1-containing JARID_1-367_ was mixed 2:1 with nucleosomes and dialyzed into 20 mM HEPES, pH 7.9, 5% v/v glycerol, 1 mM DTT for 6 h. S-adenosylmethionine was added to a final concentration of 0.1 μM and samples were incubated for 1 h at room temperature before processing using GraFix as described for the monomeric PRC2:EZH1 complexes.

Three samples were analyzed (Table 1): PRC2 in complex with AEBP2 (a), PRC2 in complex with AEBP2, and JARID2_96-367_ in a monomeric form (b) and in a dimeric form bound to nucleosome (c). The homogeneity of samples was first examined by negative-stain EM using 0.7% (v/v) uranyl formate, as described^52^. Protein preparations that showed monodispersed particles of homogeneous size and shape were used to prepare vitrified samples for cryo-EM. Vitrified grids were prepared with a Vitrobot Mark IV (Thermo Fisher Scientific) set at 100% humidity and 4 °C.

**Table 1:** Cryo-EM data collection, refinement, and validation statistics.

	#1 PRC2:EZH1–AEBP2	#2 PRC2:EZH1–AEBP2–JARID2	#3 PRC2-nuclesome complex, nucleosome	#4 PRC2-nucleosome complex, PRC2_A	#5 PRC2-nucleosome complex, PRC2_B	#6 PRC2-nucleosome complex, composite map
	(EMD-23022)	(EMD-23021)	(EMD-23026)	(EMD-23024)	(EMD-23025)	(EMD-23103)
		(PDB 7KSO)	(PDB 7KTQ)	(PDB 7KSR)	(PDB 7KTP)	
*Data collection and processing*						
Magnification	22,500	22,500	64,000	64,000	64,000	64,000
Voltage (kV)	300	300	300	300	300	300
Electron exposure (e–/Å^2^)	47	47	56.7	56.7	56.7	56.7
Defocus range (μm)	−1.5 to −3.0	−1.5 to −3.0	−1.0 to −2.5	−1.0 to −2.5	−1.0 to −2.5	−1.0 to −2.5
Pixel size (Å)	1.3	1.3	1.35	1.35	1.35	1.35
Symmetry imposed	C1	C1	C1	C1	C1	C1
Initial particle images (no.)	3,142,334	1,608,434	3,410,000	3,410,000	3,410,000	3,410,000
Final particle images (no.)	155,809	211,110	56,616	18,151	26,440	*
Map resolution (Å)	4.1	3.9	3.3	4.1	4.8	*
FSC threshold	0.143	0.143	0.143	0.143	0.143	
Map resolution range (Å)	3.9–6.6	3.7–6.4	3.1–5.8	3.7–6.4	3.7–6.4	*
*Refinement*						
Initial model used (PDB code)		5HYN, 2XU7, 2YB8, 5FXY, 5WAI	1KX5, 5WCU	Model #2	Model #2	
Model resolution (Å)		4.1	3.5	4.3	6.2	
FSC threshold		0.5	0.5	0.5	0.5	
*Model resolution range (Å)*						
Map sharpening *B* factor (Å^2^)	−135	−122	−81	−104	−120	
*Model composition*						
Non-hydrogen atoms		13,075	12,745	11,863	12,008	
Protein residues		1690	752	1524	1544	
Ligands		8	0	7	7	
B *factors (Å*^*2*^*) mean*						
Protein		95.32	31.08	124.73	191.01	
Ligand		150.31	N/A	136.69	219.75	
*R.m.s. deviations*						
Bond lengths (Å)		0.005	0.005	0.005	0.005	
Bond angles (°)		1.16	0.877	1.135	1.157	
*Validation*						
MolProbity score		1.5	1.3	1.33	1.3	
Clashscore		3.83	5.59	1.91	2.11	
Poor rotamers (%)		0.29	0	0	0	
*Ramachandran plot*						
Favored (%)		95.33	98.51	94.47	95.49	
Allowed (%)		4.67	1.49	5.53	4.51	
Disallowed (%)		0	0	0	0	

^*^Composite map comprised of #3–5, see individual maps for statistics.

For PRC2:EZH1–AEBP2 and monomeric PRC2:EZH1–AEBP2–JARID2, 4-µl aliquots at 0.05 mg/ml were applied to glow-discharged Quantifoil 300 mesh 1.2/1.3 gold grids. The grids were blotted for 3 s with a blot force setting of −2 and then plunged into liquid ethane. Before freezing in liquid ethane, grids without an additional carbon film were blotted for 3 s after a 5-s waiting time, whereas grids with an additional carbon layer were blotted for 1 s after a 20-s waiting time. Grids were screened on a Talos Arctica electron microscope (Thermo Fisher Scientific) operated at an acceleration voltage of 200 kV.

Since particles at the center of holes tended to aggregate, images were taken at the edge of the holes. Image stacks were collected with a Titan Krios electron microscope (Thermo Fisher Scientific) in the Cryo-EM Resource Center at the Rockefeller University operated at an acceleration voltage of 300 kV. Data were collected at a nominal magnification of ×22,500 (calibrated pixel size of 1.3 Å on the specimen level) with a K2 Summit camera (Gatan) in super-resolution counting mode. Exposures of 10 s were dose-fractionated into 40 frames (250 ms per frame), with a dose rate of 8 electrons/pixel/s (~1.18 electrons/Å^2^/frame), resulting in a total dose of 47 electrons/Å^2^. The images were recorded with SerialEM 3.8^53^, using defocus values ranging from −1.5 to −3 µm.

For the dimeric PRC2:EZH1–AEBP2–JARID2 complex bound to core nucleosomes containing H3K27M and H2AUb modifications, samples at 0.12 mg/ml were applied to glow-discharged Quantifoil 200 mesh 1.2/1.3 gold grids. The grids were blotted for 3 s with a blot force setting of −1 and then plunged into liquid ethane. The image stacks were collected on a Titan Krios electron microscope in the Pacific Northwest Cryo-EM Center at a calibrated pixel size of 1.35 Å on the specimen level with a K3 camera in counting mode. Exposures of 10 s were dose-fractionated into 50 frames (200 ms per frame), with a dose rate of 0.84 electrons/pixel/frame, resulting in a total dose of 56.7 electrons/Å^2^.

### Cryo-EM image processing

Image stacks recorded in super-resolution mode were binned over 2 × 2 pixels. All image stacks were motion-corrected, dose-weighted, and summed in Motioncorr2^54^ (Supplementary Figs. 1a, 2a). The CTF parameters were determined with Ctffind4^55^. The particles were autopicked with Gautomatch (http://www.mrc-lmb.cam.ac.uk/kzhang/Gautomatch/) using templates obtained by 2D classification of small datasets (~3000 particles) of manually picked particles. All subsequent image-processing steps, including 2D and 3D classification, refinement, postprocessing, and local resolution estimation, were performed in RELION-2.1^56^.

For the PRC2:EZH1–AEBP2 complex, 3,142,334 particles were autopicked from 5527 micrographs. The particles were extracted into 180 × 180-pixel images and subjected to 2D classification. The particles in the classes that showed the most detailed averages were used to calculate an initial map of the PRC2:EZH1–AEBP2 complex in cryoSPARC v2^57^. After removing classes whose averages showed ice contamination or edges of the carbon film, the remaining 2,629,432 particles were subjected to 3D classification into ten classes using the map generated by cryoSPARC as the initial reference map. One class showed secondary structure. This map and four additional maps generated from it (the EED1–EZH1 sub-complex by itself, centered and off-center, and the RBAP48–SUZ12–AEBP2 sub-complex by itself, centered and off-center) were used as reference maps for a supervised 3D classification. The 677,847 particles assigned to the full PRC2:EZH1–AEBP2 complex were then subjected to unsupervised 3D classification into six classes using as reference map the same map that was used to generate the references for the supervised classification (Supplementary Fig. 1c, green map). The 245,208 particles in the two classes showing the most detail were combined and subjected to another round of 3D classification into six classes. The 155,809 particles in the four classes showing the most detailed structure were combined and refined, yielding a map at 4.1-Å resolution after postprocessing (Supplementary Fig. 3d).

For the monomeric PRC2:EZH1–AEBP2–JARID2 complex, a total of 1,608,434 particles were autopicked from 4831 micrographs. The particles were extracted into 180 × 180-pixel images and subjected to 2D classification. Compared with the PRC2:EZH1–AEBP2 sample, a larger number of averages showed the intact complex, suggesting that JARID2 stabilizes the complex. After removing classes representing ice contamination and carbon edges as well as classes whose averages showed no features, the remaining 1,025,945 particles were subjected to 3D classification into eight classes. The 236,094 particles in the class showing the most structural detail were subjected to the second round of 3D classification into six classes. The 211,110 particles in the five classes showing the most detailed structure were combined and refined, yielding a map at 3.9-Å resolution after postprocessing. To further improve the quality of the map, the 236,094 particles from the best class resulting from the first 3D classification were combined with the 245,208 particles from the best two classes resulting from the first unsupervised 3D classification of the PRC2:EZH1–AEBP2 dataset and subjected to 3D classification into six classes. The 329,184 particles from the four classes showing the most detailed structure were combined and refined, yielding a map at 3.9-Å resolution after postprocessing (Supplementary Fig. 2c). The Bayesian polishing and CTF refinement procedures implemented in RELION-3.1 did not improve map quality.

For the dimeric PRC2:EZH1–AEBP2–JARID2 complex bound to core nucleosomes containing H3K27M and H2AUb modifications, a total of 7658 movies were collected at the Pacific Northwest Cryo-EM Center using a Titan Krios and K3 camera (Supplementary Fig. 5a). Patch motion correction and patch CTF correction were done in cryoSPARC v2^57^. In total, 3,410,00 particles were autopicked from 7384 aligned micrographs using references generated from a subset of ~1000 manually picked particles. The particles were extracted into 300 × 300-pixel images and subjected to multiple rounds of 2D classification in cryoSPARC resulting in 742,000 particles (Supplementary Fig. 5b). cryoSPARC was used to generate an ab initio map that was subsequently used in multiple rounds of heterogeneous refinement. Classes containing stronger density for two PRC2 complexes bound to a nucleosome were selected and used as input particles for additional heterogenous refinements until no further classification was observed. A stack of 109,858 particles was imported into RELION-3.0^58^ for final processing (Supplementary Fig. 5c). Signal subtraction and refinements using masks were employed to generate initial maps of the nucleosome and each monomer of PRC2 individually. Particles from each component were subjected to 3D classification without alignment, and the best classes were used to refine each component individually (Supplementary Fig. 5d). In total, 56,616 particles yielded a map of the nucleosome at 3.3-Å resolution after postprocessing, 18,151 particles yielded a map of PRC2:EZH1–AEBP2–JARID2 complex “A” at 4.1-Å resolution, and 26,440 particles yielded a map of PRC2:EZH1–AEBP2–JARID2 complex “B” at 4.8-Å resolution (Supplementary Fig. 6a–c).

### Model building and refinement of the monomeric PRC2

Initial fitting of subunits Ezh1, EED, RBAP48, and VEFS domain of SUZ12: PDB: 5HYN was used to model EZH1, EED, and SUZ12_561-686_ (VEFS). PDB: 2XU7 was used for modeling RBAP48^27,59^. The coordinates of each subunit were individually fit into the density using UCSF Chimera’s v1.14 “Fit in map” function^60^. Coot v0.8 was used for manual adjustment of domains, secondary-structure elements, and side chains into densities^61^. The complete model was refined using real-space refinement in PHENIX v1^62^. The EZH2 model in PDB: 5HYN was manually replaced with a sequence from EZH1 where applicable. Additional portions were added from an in silico EZH1 model prediction (Swiss-Model: Q92800)^63^. Zinc atoms were rigid-body fit into the appropriate density based on PDB: 5HYN.

#### SUZ12 model

The “neck” region (aa 494–562), Zinc finger (aa 362–495), and ZnB/WDB1 (aa 78–146) of SUZ12 were built using models from PDB: 2YB8 and PDB: 5FXY and manual building from scratch in Coot based on the secondary-structure predictions from PSIPRED v3.3 and using aromatic residues as anchor points^31,32^. A crystal structure of the lower lobe (PDB: 5WAI) was used as a starting model for the rest of SUZ12, followed by manual building in Coot^29^.

#### AEBP2 and JARID2 models

The AEBP2 model was built based on PDB: 5WAI^28,29^. Residues 391–418 of AEBP2 contributing to a lower lobe protein–protein interaction helix bundle were manually built in Coot by using a dummy α-helix chain and using the aromatic residues in the sequence as anchor points. Residues 149–177 of JARID2 were modeled based on PDB: 5WAI and manual fitting to the cryo-EM density in Coot.

#### Refinement of PRC2:EZH1

After local adjustments of secondary-structure elements and side chains into densities in Coot, the complete model was refined in real-space (PHENIX) using secondary structure, rotamer, and Ramachandran restraints in 100 iterations. It was then visually inspected, manually adjusted where appropriate, and evaluated for Ramachandran outliers. Figures were prepared using Chimera v1.14^60^, ChimeraX v1.0^64^, and PyMol v2 (Schrödinger).

### Size-exclusion chromatography and multiangle light scattering

A TSKgel G4000SW_xl_ HPLC column (TOSOH) was equilibrated in MALS buffer (20 mM Tris-HCl, pH 7.5, 300 mM NaCl, and 1 mM DTT) at 0.5 ml/min using a Waters HPLC system. PRC2 complexes were diluted to 1.25 mg/ml in MALS buffer and 30 μl samples were injected onto the column. Multiangle light scattering data were collected using a miniDAWN and Optilab rEX, and analyzed using ASTRA (Wyatt).

### Chromatin-compaction assay

Chromatin-compaction experiments were based on the phase-separation assays in Gibson et al.^37^. A fragment of DNA containing twelve 601 nucleosome-positioning sequences with 187 base pairs (12_187_601) nucleosome repeat length was prepared as described above for single 601 nucleosome-positioning constructs^47^. *Xenopus laevis* histone H2B containing a single mutant cysteine residue (120C) was reduced by dissolving the lyophilized protein in Reducing Buffer (RB) (20 mM Tris-HCl, pH 7.5, 6 M guanidine HCl, 5 mM EDTA, 75 mM β-mercaptoethanol). After 90 min at room temperature, the histones were transferred into RB without β-mercaptoethanol using a HiTrap 5-ml desalting column. A 5:1 molar excess of fluorescein-5-maleimide (Molecular Probes) dissolved in dimethylsulfoxide (DMSO) was added to the reduced histones and incubated at room temperature overnight. Free dye was removed by extensive dialysis into unfolding buffer (6 M guanidine HCl, 20 mM Tris-HCl, pH 7.5, 5 mM DTT). Labeled and unlabeled histones were mixed at a 0.5:0.5:1:1:1 ratio of labeled H2B:unlabeled H2B:H2A:H3:H4 in unfolding buffer and assembled into octamers as described above. Labeled octamers were mixed 1:10 with unlabeled octamers and used to assemble nucleosome arrays with 12_187_601.

PRC2 samples were extensively dialyzed into phase-separation buffer (PSB) (25 mM Tris-HCl, pH 7.5, 150 mM KCl, 0.1 mM EDTA, 10% v/v glycerol, 5 mM DTT). Labeled chromatin at 12 μM nucleosomes in Tris-EDTA buffer (TE) (10 mM Tris pH 8.0, 0.1 mM EDTA, 1 mM DTT) was diluted 1:1 to 6 μM using 2× chromatin dilution buffer (50 mM Tris-HCl, pH 7.5, 0.2 mM EDTA, 0.4 mg/ml BSA, 10 mM DTT). Serial dilutions of PRC2 complexes were made in PSB, and then an equal volume of chromatin was mixed with PRC2 to induce phase separation. Reactions were incubated at room temperature for 30 min, then imaged on a Zeiss 880 confocal microscope using Zen 2.5 (Zeiss).

### Reporting summary

Further information on research design is available in the Nature Research Reporting Summary linked to this article.

## Supplementary information

Supplementary Information

Reporting Summary

Description of Additional Supplementary Files

Supplementary Movie 1

## Data Availability

Data supporting the findings of this paper are available from the corresponding authors upon reasonable request. The Cryo-EM density maps of the PRC2:EZH1–AEBP2: EMD-23022, PRC2:EZH1–AEBP2–JARID2: EMD-23021, PRC2–nucleosome (nucleosome): EMD-23026, PRC2–nucleosome (PRC2_A): EMD-23024, PRC2–nucleosome (PRC2_B): EMD-23025 and PRC2 nucleosome (composite map): EMD-23103 complexes have been deposited in the Electron Microscopy Data Bank. The atomic coordinates for PRC2:EZH1–AEBP2–JARID2: 7KSO, PRC2–nucleosome (nucleosome): 7KTQ, PRC2–nucleosome (PRC2_A): 7KSR, and PRC2–nucleosome (PRC2_B): 7KTP complexes have been deposited in the RCSB Protein Data Bank. Source data are provided with this paper.

## Source data

Source Data

## References

[1] Lewis EB (1978). A gene complex controlling segmentation in Drosophila. Nature.

[2] Grossniklaus U, Paro R (2014). Transcriptional silencing by polycomb-group proteins. Cold Spring Harb. Perspect. Biol..

[3] Kuzmichev A, Nishioka K, Erdjument-Bromage H, Tempst P, Reinberg D (2002). Histone methyltransferase activity associated with a human multiprotein complex containing the enhancer of Zeste protein. Genes Dev..

[4] Muller J (2002). Histone methyltransferase activity of a Drosophila Polycomb group repressor complex. Cell.

[5] Ciferri C (2012). Molecular architecture of human polycomb repressive complex 2. eLife.

[6] Cao R (2002). Role of histone H3 lysine 27 methylation in polycomb-group silencing. Science.

[7] Yu JR, Lee CH, Oksuz O, Stafford JM, Reinberg D (2019). PRC2 is high maintenance. Genes Dev..

[8] Hansen KH (2008). A model for transmission of the H3K27me3 epigenetic mark. Nat. Cell Biol..

[9] Margueron R (2009). Role of the polycomb protein EED in the propagation of repressive histone marks. Nature.

[10] Oksuz O (2018). Capturing the onset of PRC2-mediated repressive domain formation. Mol. Cell.

[11] Lee CH (2018). Allosteric activation dictates PRC2 activity independent of its recruitment to chromatin. Mol. Cell.

[12] Lewis PW (2013). Inhibition of PRC2 activity by a gain-of-function H3 mutation found in pediatric glioblastoma. Science.

[13] Stafford, J. M. et al. Multiple modes of PRC2 inhibition elicit global chromatin alterations in H3K27M pediatric glioma. *Sci. Adv.* **4**, ARTN eaau5935 (2018). 10.1126/sciadv.aau5935 PMC6209383 30402543

[14] Diehl KL (2019). PRC2 engages a bivalent H3K27M-H3K27me3 dinucleosome inhibitor. Proc. Natl Acad. Sci. USA.

[15] Cao R, Zhang Y (2004). SUZ12 is required for both the histone methyltransferase activity and the silencing function of the EED-EZH2 complex. Mol. Cell.

[16] Li G (2010). Jarid2 and PRC2, partners in regulating gene expression. Genes Dev..

[17] Son J, Shen SS, Margueron R, Reinberg D (2013). Nucleosome-binding activities within JARID2 and EZH1 regulate the function of PRC2 on chromatin. Genes Dev..

[18] Lee CH (2018). Distinct stimulatory mechanisms regulate the catalytic activity of polycomb repressive complex 2. Mol. Cell.

[19] Laible G (1997). Mammalian homologues of the polycomb-group gene enhancer of zeste mediate gene silencing in Drosophila heterochromatin and at *S. cerevisiae* telomeres. EMBO J..

[20] Margueron R (2008). Ezh1 and Ezh2 maintain repressive chromatin through different mechanisms. Mol. Cell.

[21] Ezhkova E (2011). EZH1 and EZH2 cogovern histone H3K27 trimethylation and are essential for hair follicle homeostasis and wound repair. Genes Dev..

[22] Hidalgo I (2012). Ezh1 is required for hematopoietic stem cell maintenance and prevents senescence-like cell cycle arrest. Cell Stem Cell.

[23] Vo LT (2018). Regulation of embryonic haematopoietic multipotency by EZH1. Nature.

[24] Davidovich C, Goodrich KJ, Gooding AR, Cech TR (2014). A dimeric state for PRC2. Nucleic Acids Res..

[25] Shen X (2008). EZH1 mediates methylation on histone H3 lysine 27 and complements EZH2 in maintaining stem cell identity and executing pluripotency. Mol. Cell.

[26] Chen, S., Jiao, L., Liu, X., Yang, X. & Liu, X. A dimeric structural scaffold for PRC2-PCL targeting to CpG island chromatin. *Mol. Cell*10.1016/j.molcel.2019.12.019 (2020). 10.1016/j.molcel.2019.12.019 PMC7571800 31959557

[27] Justin N (2016). Structural basis of oncogenic histone H3K27M inhibition of human polycomb repressive complex 2. Nat. Commun..

[28] Kasinath V (2018). Structures of human PRC2 with its cofactors AEBP2 and JARID2. Science.

[29] Chen S, Jiao L, Shubbar M, Yang X, Liu X (2018). Unique structural platforms of Suz12 dictate distinct classes of PRC2 for chromatin binding. Mol. Cell.

[30] Poepsel S, Kasinath V, Nogales E (2018). Cryo-EM structures of PRC2 simultaneously engaged with two functionally distinct nucleosomes. Nat. Struct. Mol. Biol..

[31] Schmitges FW (2011). Histone methylation by PRC2 is inhibited by active chromatin marks. Mol. Cell.

[32] Millard CJ (2016). The structure of the core NuRD repression complex provides insights into its interaction with chromatin. eLife.

[33] Cooper S (2016). Jarid2 binds mono-ubiquitylated H2A lysine 119 to mediate crosstalk between polycomb complexes PRC1 and PRC2. Nat. Commun..

[34] Kalb R (2014). Histone H2A monoubiquitination promotes histone H3 methylation in polycomb repression. Nat. Struct. Mol. Biol..

[35] Valencia-Sanchez MI (2019). Structural basis of Dot1L stimulation by histone H2B lysine 120 ubiquitination. Mol. Cell.

[36] Hojfeldt JW (2018). Accurate H3K27 methylation can be established de novo by SUZ12-directed PRC2. Nat. Struct. Mol. Biol..

[37] Gibson BA (2019). Organization of chromatin by intrinsic and regulated phase separation. Cell.

[38] Sigler PB (1988). Transcriptional activation. Acid blobs and negative noodles. Nature.

[39] Grau DJ (2011). Compaction of chromatin by diverse polycomb group proteins requires localized regions of high charge. Genes Dev..

[40] Lau MS (2017). Mutation of a nucleosome compaction region disrupts polycomb-mediated axial patterning. Science.

[41] Tatavosian R (2019). Nuclear condensates of the polycomb protein chromobox 2 (CBX2) assemble through phase separation. J. Biol. Chem..

[42] Plys AJ (2019). Phase separation of Polycomb-repressive complex 1 is governed by a charged disordered region of CBX2. Genes Dev..

[43] Larson AG (2017). Liquid droplet formation by HP1alpha suggests a role for phase separation in heterochromatin. Nature.

[44] Strom AR (2017). Phase separation drives heterochromatin domain formation. Nature.

[45] Laugesen A, Hojfeldt JW, Helin K (2019). Molecular mechanisms directing PRC2 recruitment and H3K27 methylation. Mol. Cell.

[46] Weissmann F (2016). biGBac enables rapid gene assembly for the expression of large multisubunit protein complexes. Proc. Natl Acad. Sci. USA.

[47] Dyer PN (2004). Reconstitution of nucleosome core particles from recombinant histones and DNA. Methods Enzymol..

[48] Armache KJ, Garlick JD, Canzio D, Narlikar GJ, Kingston RE (2011). Structural basis of silencing: Sir3 BAH domain in complex with a nucleosome at 3.0 A resolution. Science.

[49] Lee KM, Narlikar G (2001). Assembly of nucleosomal templates by salt dialysis. Curr. Protoc. Mol. Biol..

[50] Rueden CT (2017). ImageJ2: ImageJ for the next generation of scientific image data. BMC Bioinforma..

[51] Kastner B (2008). GraFix: sample preparation for single-particle electron cryomicroscopy. Nat. Methods.

[52] Ohi M, Li Y, Cheng Y, Walz T (2004). Negative staining and image classification—powerful tools in modern electron microscopy. Biol. Proced. Online.

[53] Mastronarde DN (2005). Automated electron microscope tomography using robust prediction of specimen movements. J. Struct. Biol..

[54] Zheng SQ (2017). MotionCor2: anisotropic correction of beam-induced motion for improved cryo-electron microscopy. Nat. Methods.

[55] Rohou A, Grigorieff N (2015). CTFFIND4: Fast and accurate defocus estimation from electron micrographs. J. Struct. Biol..

[56] Kimanius, D., Forsberg, B. O., Scheres, S. H. & Lindahl, E. Accelerated cryo-EM structure determination with parallelisation using GPUs in RELION-2. *eLife* **5**, 10.7554/eLife.18722 (2016). 10.7554/eLife.18722 PMC5310839 27845625

[57] Punjani A, Rubinstein JL, Fleet DJ, Brubaker M (2017). A. cryoSPARC: algorithms for rapid unsupervised cryo-EM structure determination. Nat. Methods.

[58] Zivanov, J. et al. New tools for automated high-resolution cryo-EM structure determination in RELION-3. *eLife* **7**, 10.7554/eLife.42166 (2018). 10.7554/eLife.42166 PMC6250425 30412051

[59] Lejon S (2011). Insights into association of the NuRD complex with FOG-1 from the crystal structure of an RbAp48.FOG-1 complex. J. Biol. Chem..

[60] Pettersen EF (2004). UCSF Chimera—a visualization system for exploratory research and analysis. J. Comput. Chem..

[61] Emsley P, Lohkamp B, Scott WG, Cowtan K (2010). Features and development of Coot. Acta Crystallogr D. Biol. Crystallogr.

[62] Adams PD (2010). PHENIX: a comprehensive Python-based system for macromolecular structure solution. Acta Crystallogr D. Biol. Crystallogr.

[63] Waterhouse A (2018). SWISS-MODEL: homology modelling of protein structures and complexes. Nucleic Acids Res..

[64] Pettersen, E. F. et al. UCSF ChimeraX: structure visualization for researchers, educators, and developers. *Protein Sci.*10.1002/pro.3943 (2020). 10.1002/pro.3943 PMC7737788 32881101

