# Supplementary material for: Structures of monomeric and dimeric PRC2:EZH1 reveal flexible modules involved in chromatin compaction
Source: Nat Commun. 2021 Jan 29;12:714. doi: 10.1038/s41467-020-20775-z (PMC7846606; doi:10.1038/s41467-020-20775-z)

Supplementary data

Supplementary Fig. 1: Single-particle cryo-EM analysis of the PRC2:EZH1–AEBP2 complex.

a, Cryo-EM micrograph area of vitrified PRC2:EZH1–AEBP2 complex. Scale bar: 500 nm. **b,** 2D-class averages of the PRC2:EZH1–AEBP2 complex obtained with RELION-2.1. Side length of individual averages: 23 nm. **c,** 3D classification used to generate the reference maps for supervised 3D classification. **d,** Image-processing workflow for 3D classification and refinement in RELION-2.1 that resulted in a density map at 4.1-Å resolution. See Materials and Methods for details.

Supplementary Fig. 2: Single-particle cryo-EM analysis of the PRC2:EZH1–AEBP2–JARID2 complex.

a, Cryo-EM micrograph area of vitrified PRC2:EZH1–AEBP2–JARID2 complex. Scale bar: 500 nm. **b,** 2D-class averages of the PRC2:EZH1–AEBP2–JARID2 complex obtained with RELION-2.1. Side length of individual averages: 23 nm. **c,** Image-processing workflow for 3D classification and refinement in RELION-2.1 that resulted in two density maps at 3.9-Å resolution. See Materials and Methods for details.

Supplementary Fig. 3: Quality assessment for the PRC2:EZH1 map.

a, FSC curves calculated from independently refined half maps for the PRC2:EZH1–AEBP2 complex (blue), the monomeric PRC2:EZH1–AEBP2–JARID2 complex (green), and the combined dataset of the PRC2:EZH1–AEBP2 and monomeric PRC2:EZH1–AEBP2–JARID2 complexes (orange). **b,** The local resolution map for the map obtained with the combined datasets of the PRC2:EZH1–AEBP2 and monomeric PRC2:EZH1–AEBP2–JARID2 complexes. **c,** FSC comparison of the model of the monomeric PRC2:EZH1 with density maps. The combined map was used for model building, but only half map 1 was used for model refinement. **d,** Left: 3.9-Å resolution cryo-EM map of PRC2:EZH1 showing the upper and lower lobes. Right: Model built into the 3.9-Å resolution map. Subunits are colored and indicated. **e,** Representative cryo-EM densities for the 3.9-Å resolution map obtained with the combined datasets of the PRC2:EZH1–AEBP2 and monomeric PRC2:EZH1–AEBP2–JARID2 complexes. Top row, from left to right: EED, residues 170-176; EED, residues 183-191; EED, residues 193-200; SUZ12, residues 561-568; SUZ12, residues 589-602; RBAP48, residues 6-29. Bottom row, left to right: RBAP48, residues 396-404; RBAP48, residues 376-383; RBAP48, residues 31-40; RBAP48, residues 319-326; AEBP2, residues 399-418; SUZ12, residues 97-107. **f,** Comparison of the AEBP2 structure from our monomeric PRC2:EZH1–AEBP2–JARID2 (purple) complex with PDB: 6C24 (cyan) and PDB: 5WAI (green). **g,** Fit of our AEBP2 model into the cryo-EM density.

Supplementary Fig. 4: Regulatory domains of EZH1 are flexible.

a, Cartoon representation of domains that are visible in the cryo-EM density of EZH1. **b,** Depiction of the key unstructured loops in EZH1. Labels indicate the last structured residues flanking the flexible loops in EZH1. **c,** Sequence comparison of the flexible loops in EZH1 and EZH2. Underlined sequences represent regions rich in basic (blue) or acidic residues (red), RNA-interaction domains (purple), and automethylation sites (green). Acidic

or basic regions are based on manual inspection of the EZH1/2 sequences. RNA-interaction domains and automethylation sites are based on reports describing EZH2 features[63-66].

Supplementary Fig. 5: Single-particle cryo-EM analysis of the PRC2:EZH1–nucleosome complex.
a, Cryo-EM micrograph area of vitrified PRC2:EZH1–nucleosome complex. Scale bar: 500 nm. **b,** 2D-class averages of the PRC2:EZH1–nucleosome complex obtained with cryoSPARC. Side length of individual averages: 42 nm. **c,** Signal subtraction was used to isolate components of the PRC2:EZH1–nucleosome complex for further refinement in RELION-3.0. **d,** 3D classification without alignment of signal-subtracted particles. Number of particles in each class and estimated resolution of the maps are indicated. Boxes indicate classes that were chosen for final refinement and postprocessing in RELION-3.0.

Supplementary Fig. 6: Quality assessment for the PRC2:EZH1 component maps.
a, FSC curve calculated from independently refined half maps for PRC2A (left panel) and local resolution map (right panel). **b,** FSC curve calculated from independently refined half maps for PRC2B (left panel) and local resolution map (right panel). **c,** FSC curve calculated from independently refined half maps for nucleosome (left panel) and local resolution map (right panel). **d,** Overall (left panel) and zoomed-in view (right panel) of histone H3 tail interaction with EZH1 SET domain. **e,** Overall (left panel) and zoomed-in view (right panel) of proposed site of SUZ12 helix/DNA interaction. SUZ12 amino acids 80-107 comprising an α-helix are colored in magenta.

Supplementary Fig. 7: Specificity of PRC2:EZH1$_{5RtoA}$ mutant complex.
a, Nucleosome-binding activity of 4-component PRC2:EZH1 complexes (EZH1, SUZ12, EED, and RBAP48) containing the indicated version of EZH1. Left panel: wild-type EZH1, middle panel: R31,64,100,321,443A mutant EZH1 (5 random R to A), right panel: R360-364A mutant EZH1 (5RtoA). All reactions contain 25 nM core nucleosomes and the indicated amount of PRC2:EZH1. Reactions were repeated twice and gave similar results. **b,** Methyltransferase activity of indicated concentrations of PRC2:EZH1 complexes. Each data point is the average of three replicates and is shown as mean $\pm$ standard deviation.

Supplementary Fig. 8: Comparison of PRC2 dimer interfaces seen by cryo-EM and X-ray crystallography.
a, View of the C2 domain–RBAP48 interaction in our structure of the PRC2:EZH1 monomer. **b,** View of the "domain swapped" C2 domain–RBAP48 interaction in our structure of the nucleosome-bound PRC2:EZH1 dimer. **c,** Comparison of the PRC2:EZH1 dimerization interface in our structure of the nucleosome-bound PRC2:EZH1 dimer (orange and green) with that in the crystal structure of the PRC2 lower lobe (PDB: 6NQ3; grey). Note that for clarity only the C2 and RBAP48 domains are shown from our structure. RBAP48_A from 6NQ3 is aligned to RBAP48_A from our structure. Note that the C2_A domains pivot approximately 37° using the basic loop as an anchor. Note that the RBAP48_B domains are rotated relative to each other.

Supplementary Fig. 9: Purification of PRC2:EZH1 monomers and dimers.

a, Gel-filtration profile of PRC2:EZH1 demonstrating separation of dimer and monomer peak fractions. **b,** SEC-MALS of a mixture of PRC2:EZH1 dimers and monomers. Expected and observed sizes for the PRC2:EZH1 fractions are indicated. **c,** Negative-stain EM 2D-class averages of the PRC2:EZH1 dimer peak obtained with RELION-3.0. Side length of individual averages: 58 nm. **d,** Gel-filtration profiles of PRC2:EZH1 (left panel), PRC2:EZH1 dimer peak reinjected after a 24-hr incubation at 4°C (middle panel), and PRC2:EZH1 monomer peak reinjected after a 24-hr incubation at 4°C (right panel).

Supplementary Fig. 10: Biochemical activity of PRC2:EZH1 monomers and dimers.

a, Gel-filtration profiles and SDS-PAGE gels of PRC2:EZH1 with wild-type or mutant SUZ12 containing amino acids 193-197 mutated to alanine ($SUZ12_{193\text{-}197}$). Elution volumes are indicated in parentheses. Results were repeated with an independent preparation of mutant SUZ12 and gave similar results. **b,** Nucleosome-binding activity of the peak fractions from panel a. All reactions contain 12.5 nM core nucleosomes and the indicated amount of PRC2:EZH1. Reactions were repeated twice and gave similar results. **c,** Bioluminescence-based methyltransferase assay (MTase-Glo) showing relative activity of indicated PRC2:EZH1 complexes. Each data point is the average of three replicates and is shown as mean ± standard deviation. **d,** Confocal micrographs of serial 2-fold dilutions of PRC2:EZH1 mixed with nucleosome arrays showing the compaction of chromatin into droplets. Scale bar: 100 μm. Molarity is based on the expected size of PRC2:EZH1 monomers. All samples contain 3 μM total nucleosomes, assembled into arrays with 12 nucleosomes spaced 40 bp apart. Reactions were repeated twice with independent samples and gave similar results.

Supplementary Fig. 11: Comparison of PRC2:EZH1/2 dimers.

a, Gel-filtration profiles of PRC2:EZH1–AEBP2–JARID2 or PRC2:EZH2–AEBP2–JARID2 with dimer and monomer peaks labeled. **b,** Confocal micrographs of serial 2-fold dilutions of PRC2:EZH1 and PRC2:EZH2 dimers mixed with nucleosome arrays showing compaction of chromatin into droplets. Scale bar: 100 μm. Molarity is based on the expected size of PRC2:EZH1/2 monomers. All samples contain 3 μM total nucleosomes, assembled into arrays with 12 nuclesomes spaced 40 bp apart. Reactions were repeated three times with independent samples and gave similar results.

Supplementary Fig. 1: Single-particle cryo-EM analysis of the PRC2:EZH1–AEBP2 complex.

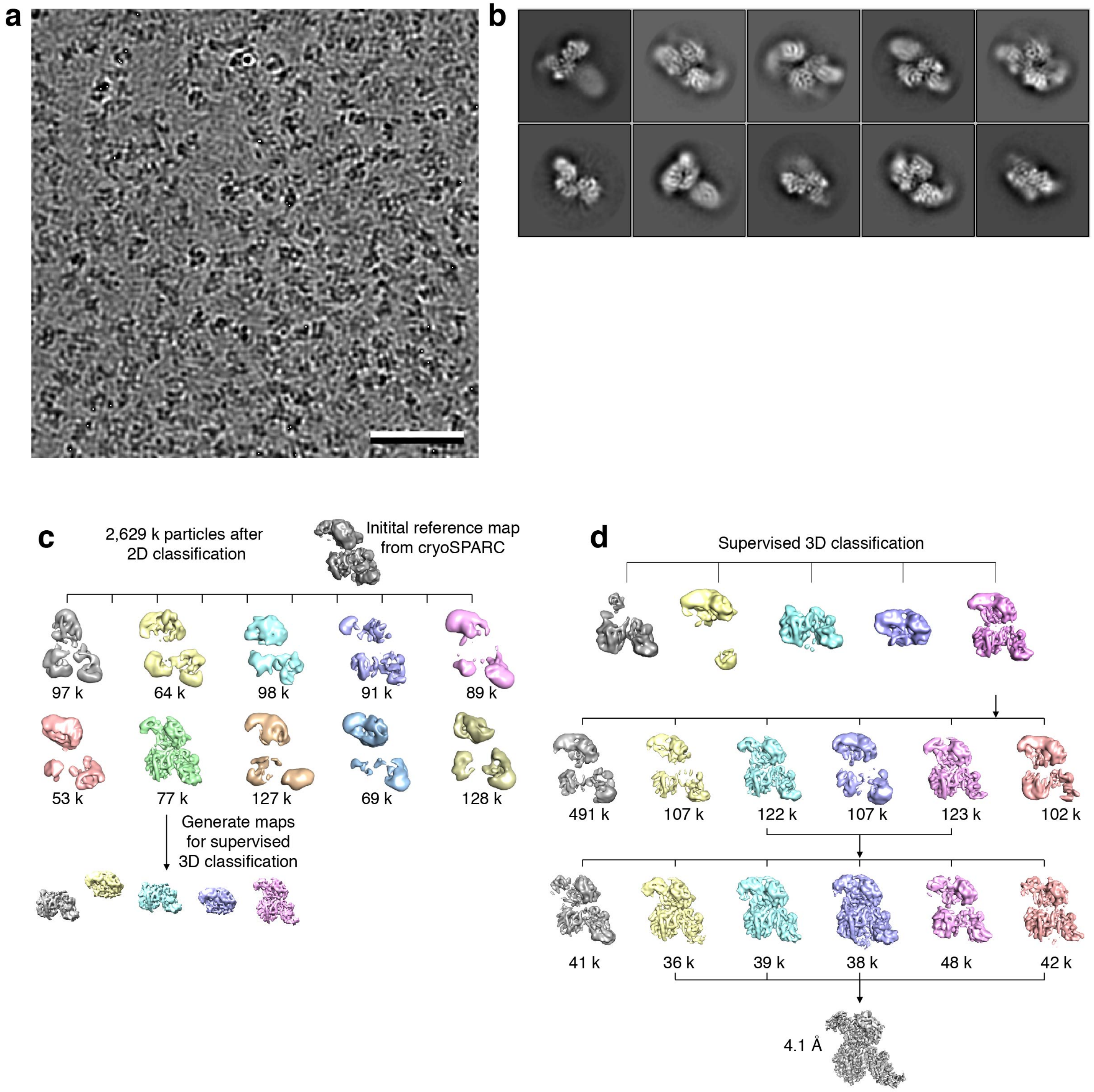

Supplementary Fig. 2: Single-particle cryo-EM analysis of the PRC2:EZH1–AEBP2–JARID2 complex.

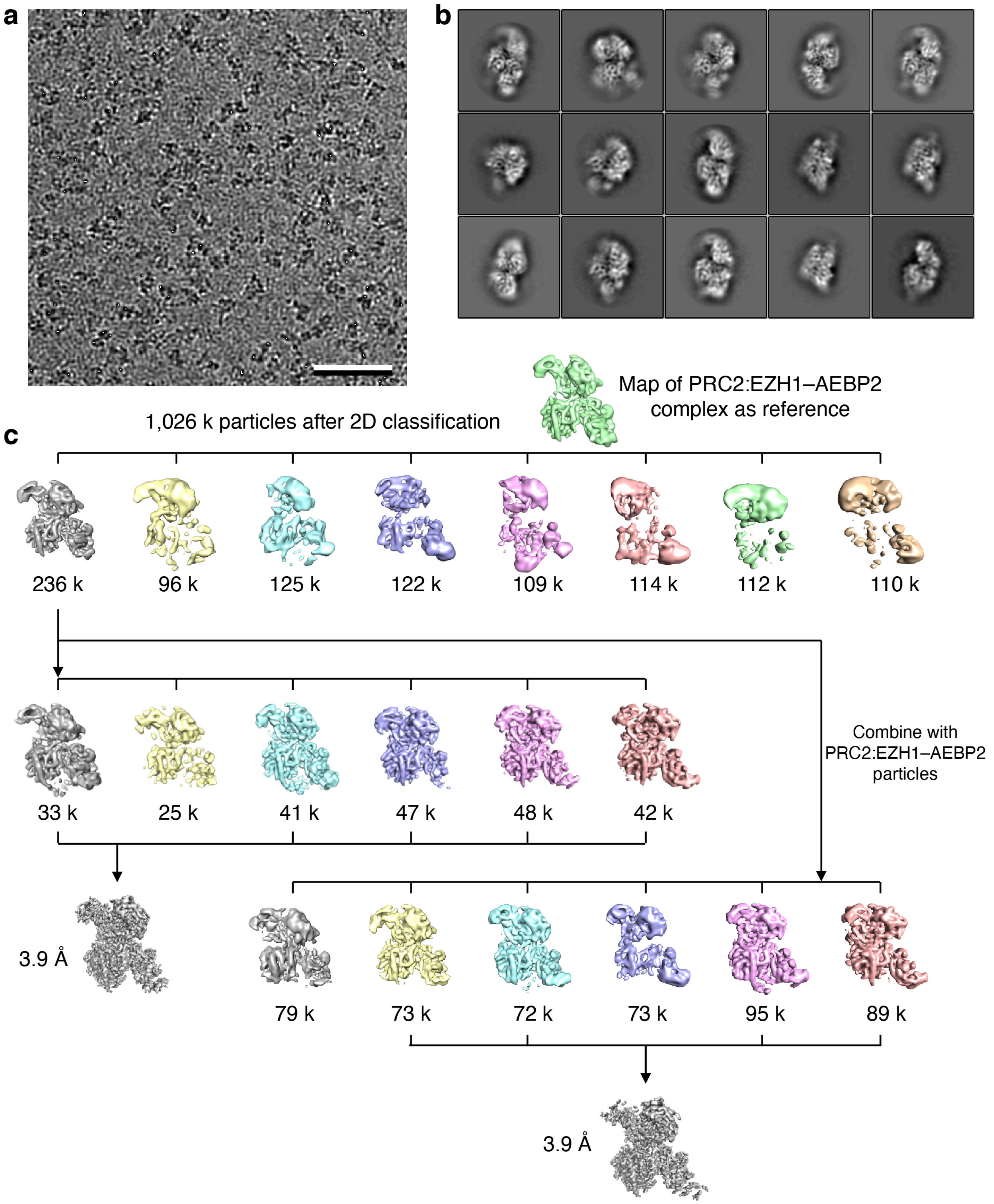

Supplementary Fig. 3: Quality assessment for the PRC2:EZH1 map.

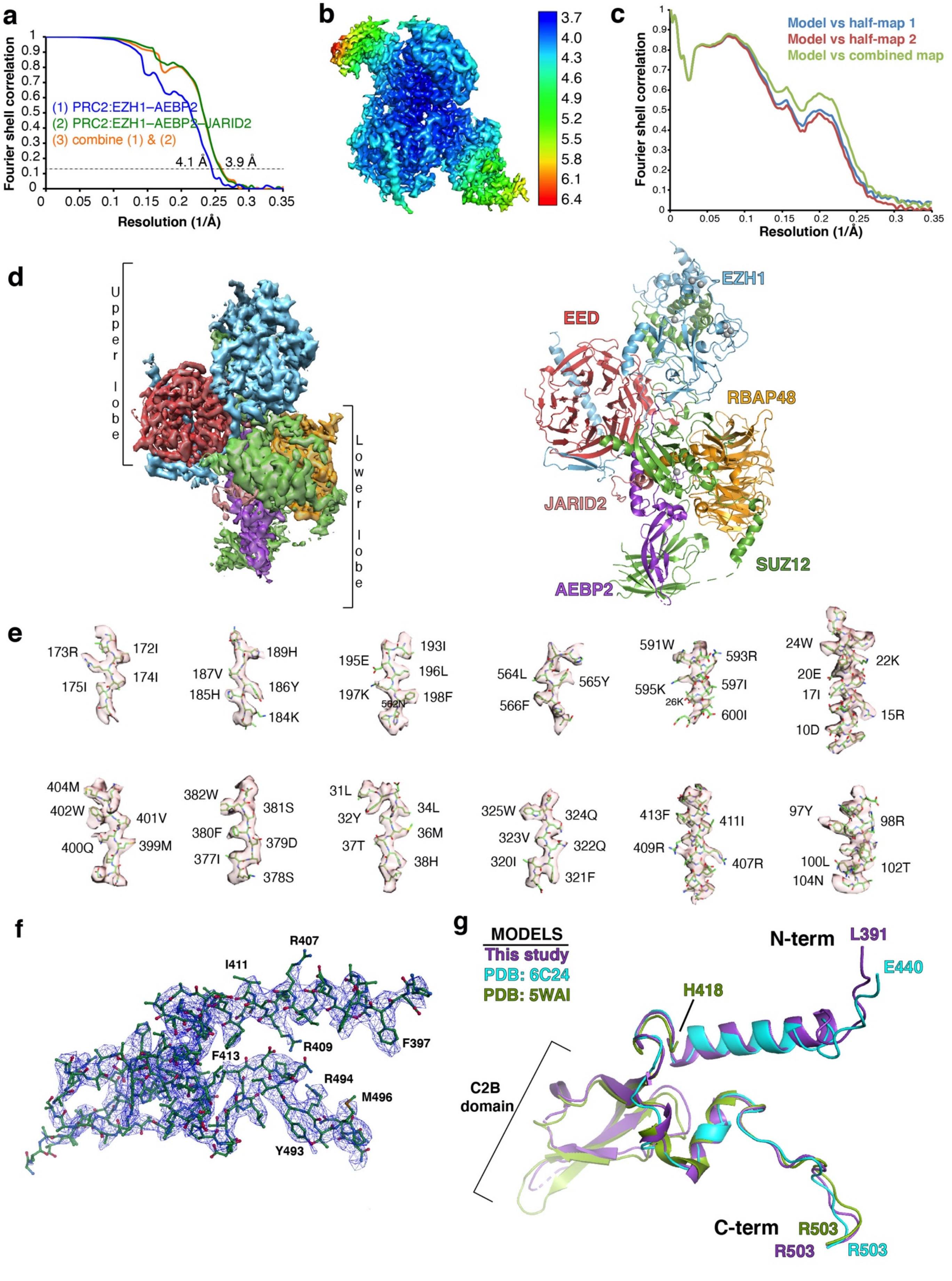

Supplementary Fig. 4: Regulatory domains of EZH1 are flexible.

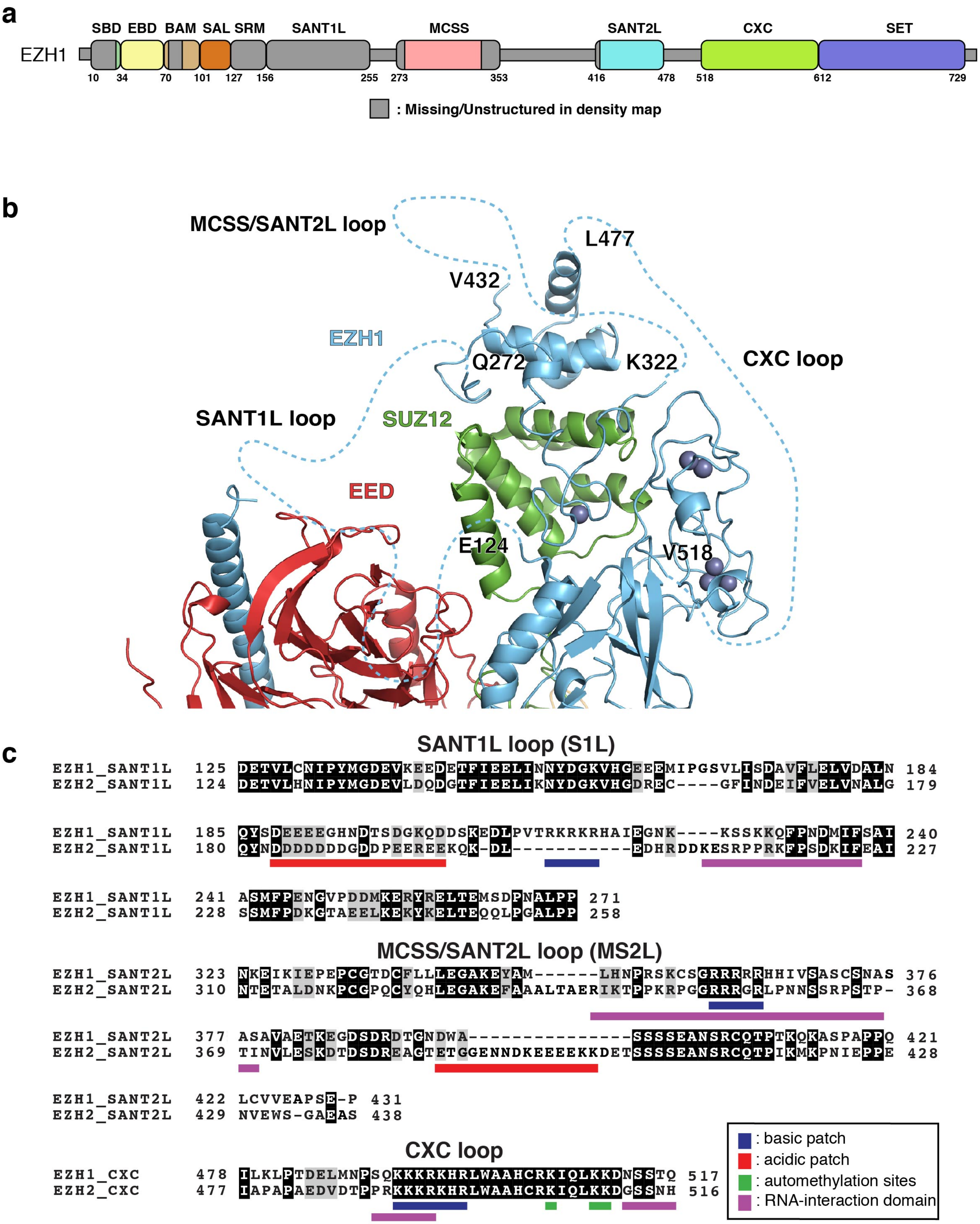

Supplementary Fig. 5: Single-particle cryo-EM analysis of the PRC2:EZH1–nucleosome complex.

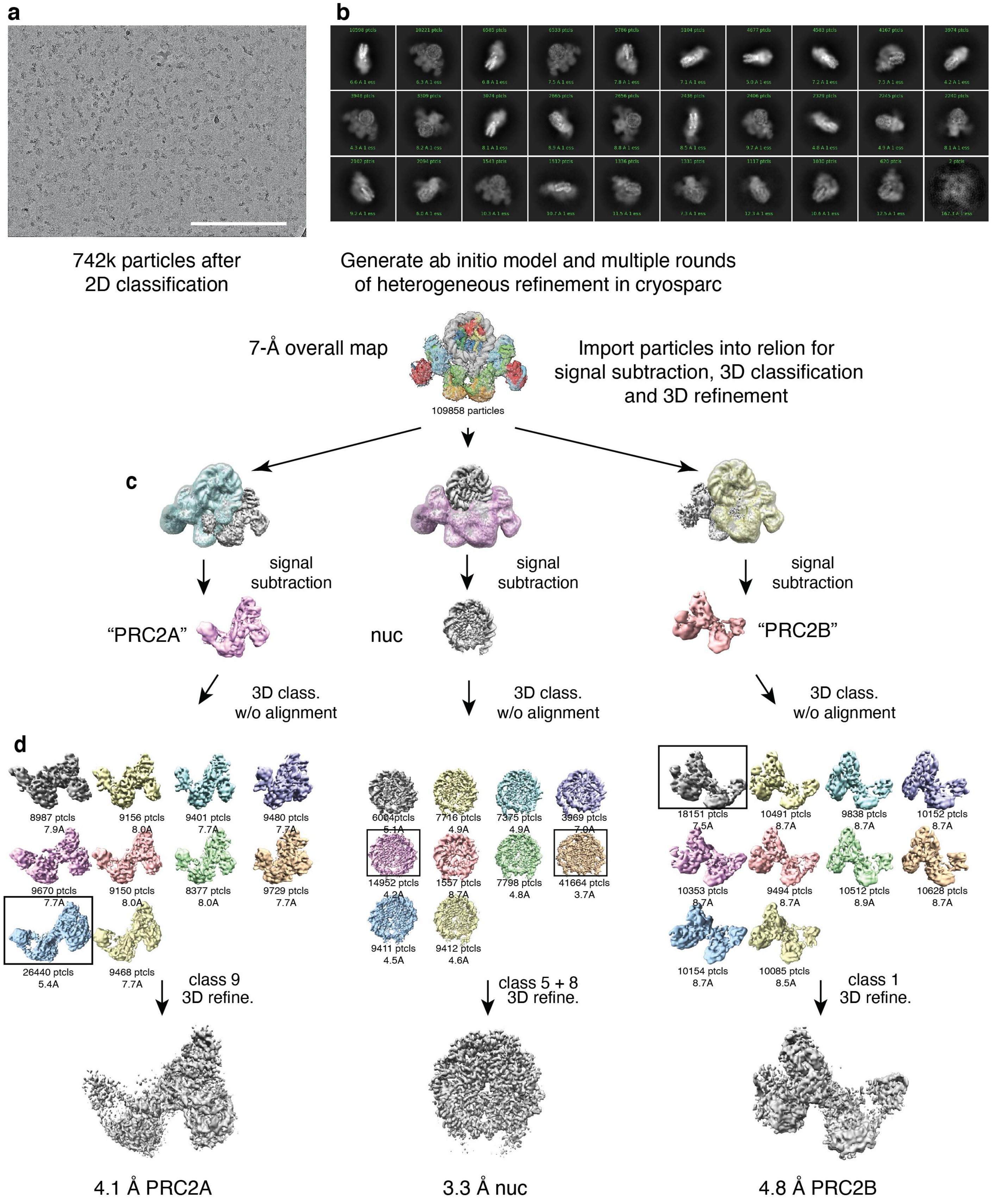

Supplementary Fig. 6: Quality assessment for the PRC2:EZH1 component maps.

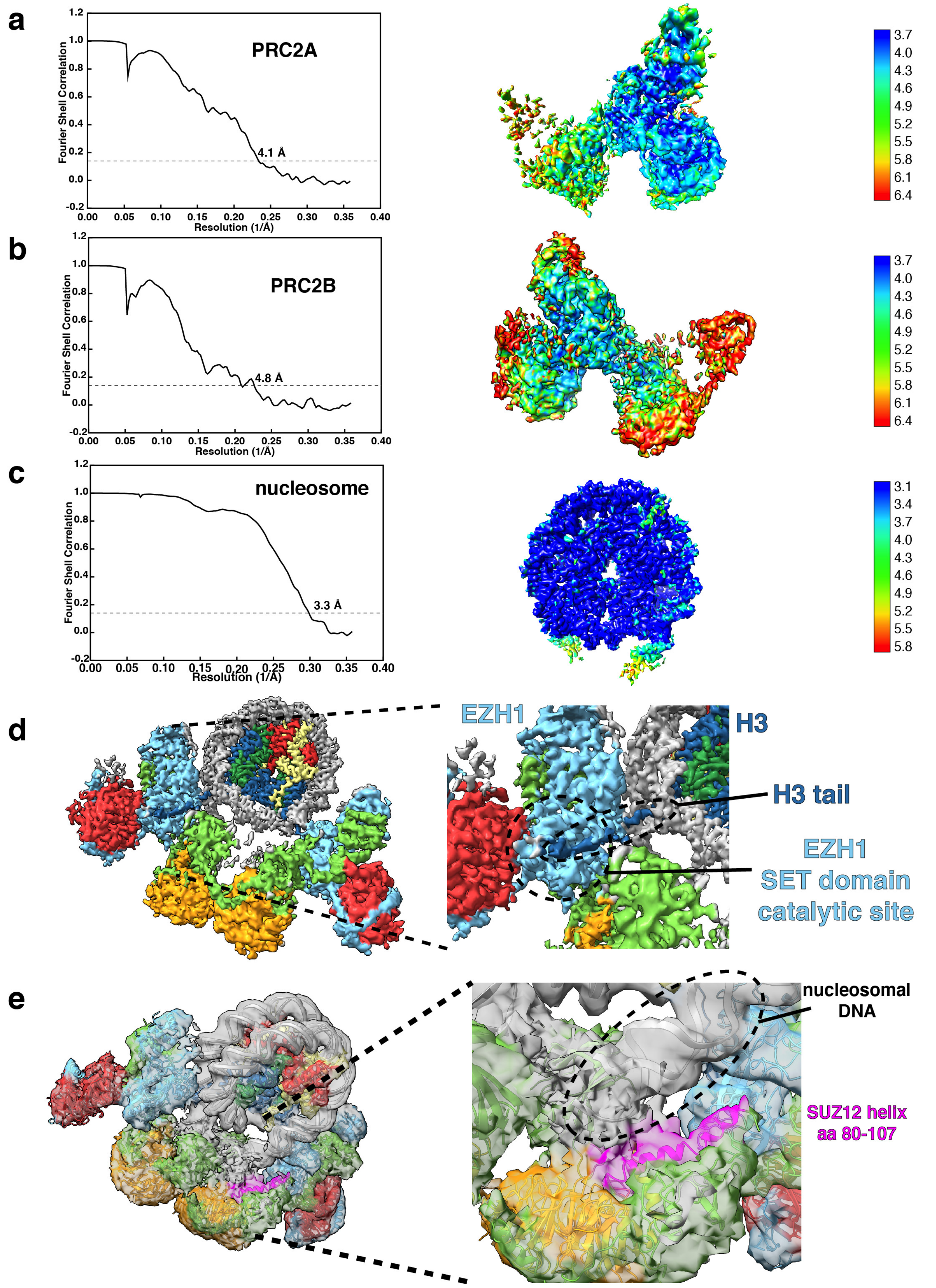

Supplementary Fig. 7: Specificity of PRC2:EZH1$_{5RtoA}$ mutant complex.

a

Nucleosome-binding activity of PRC2:EZH1

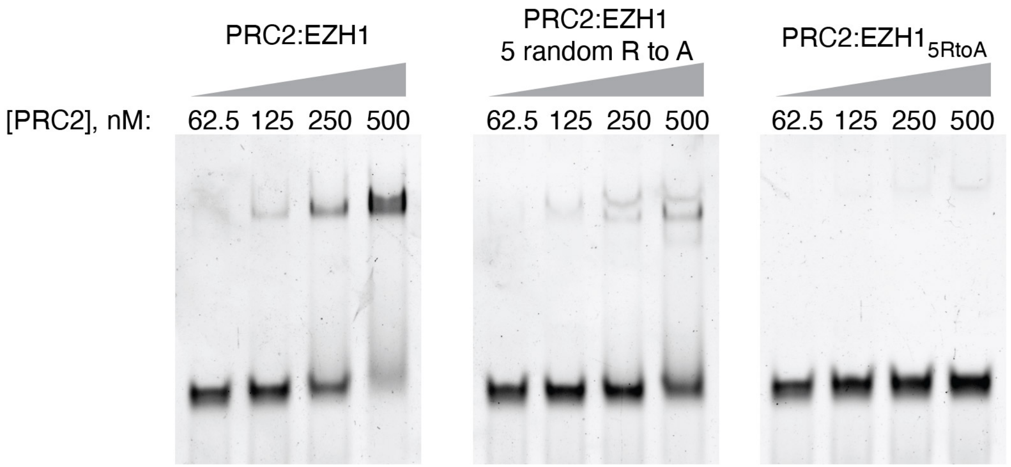

Methyltransferase activity

b

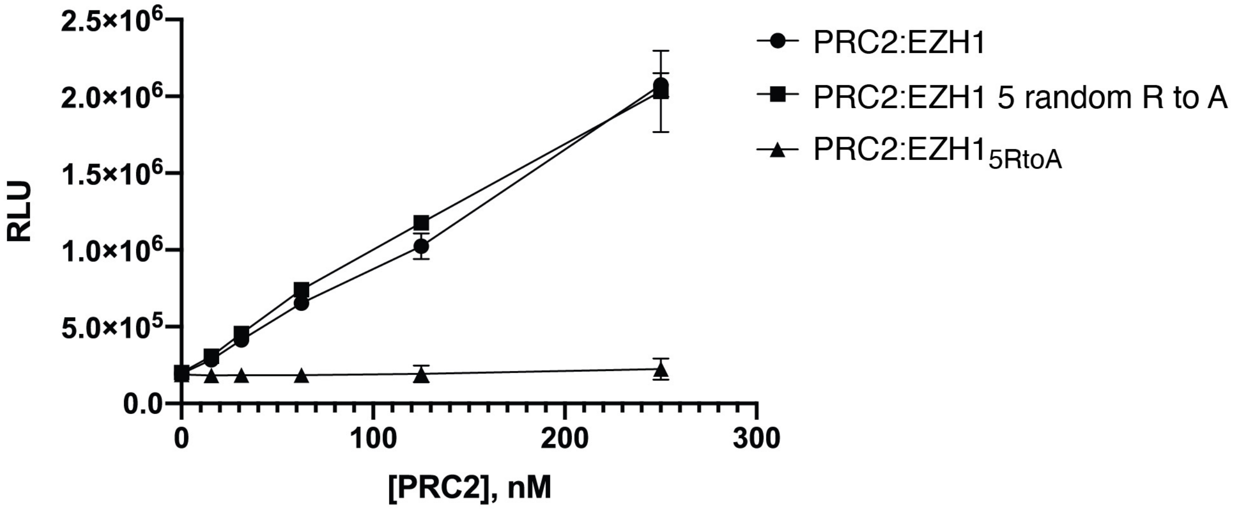

Supplementary Fig. 8: Comparison of PRC2 dimer interfaces seen by cryo-EM and X-ray crystallography.

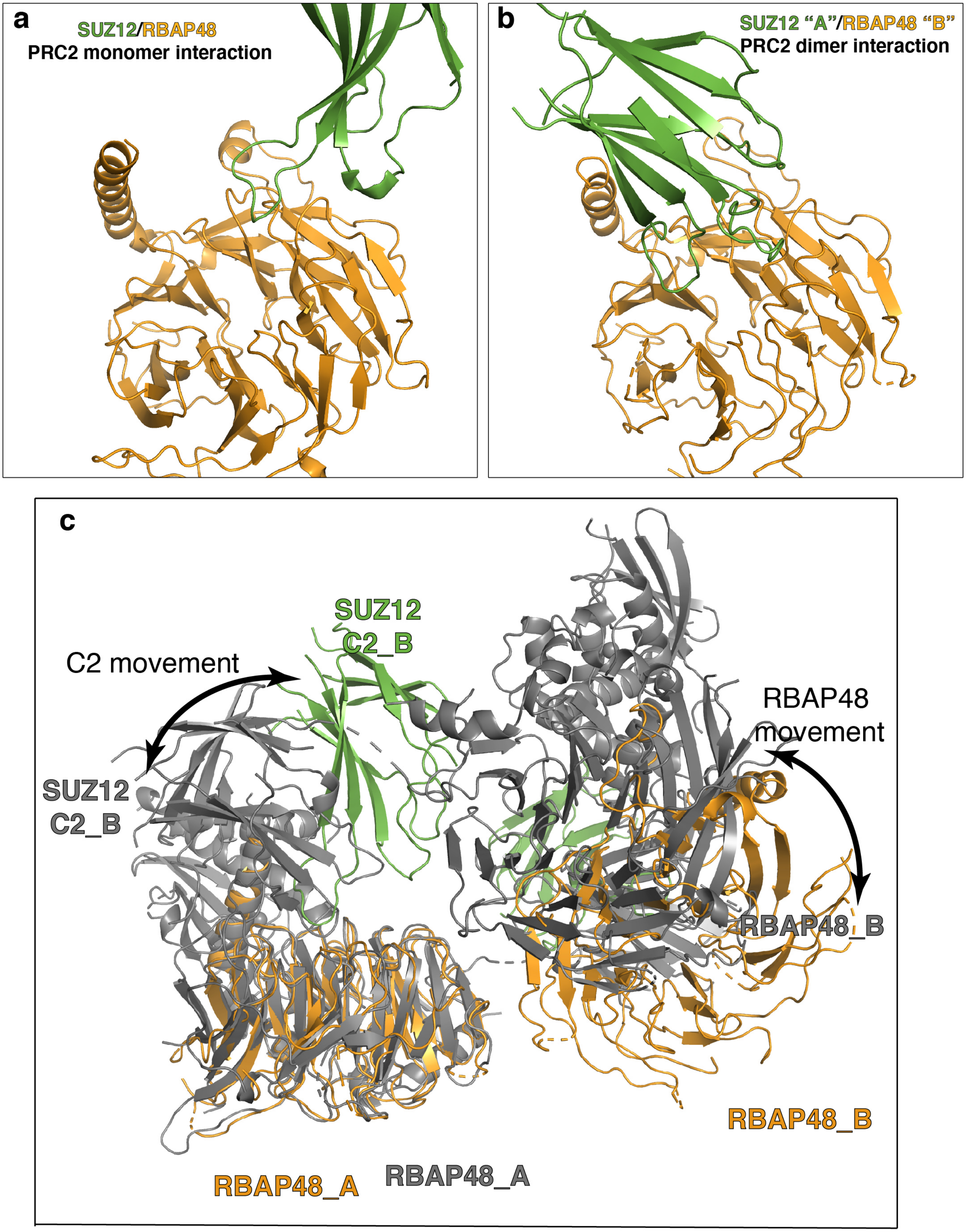

Supplementary Fig. 9: Purification of PRC2:EZH1 monomers and dimers.

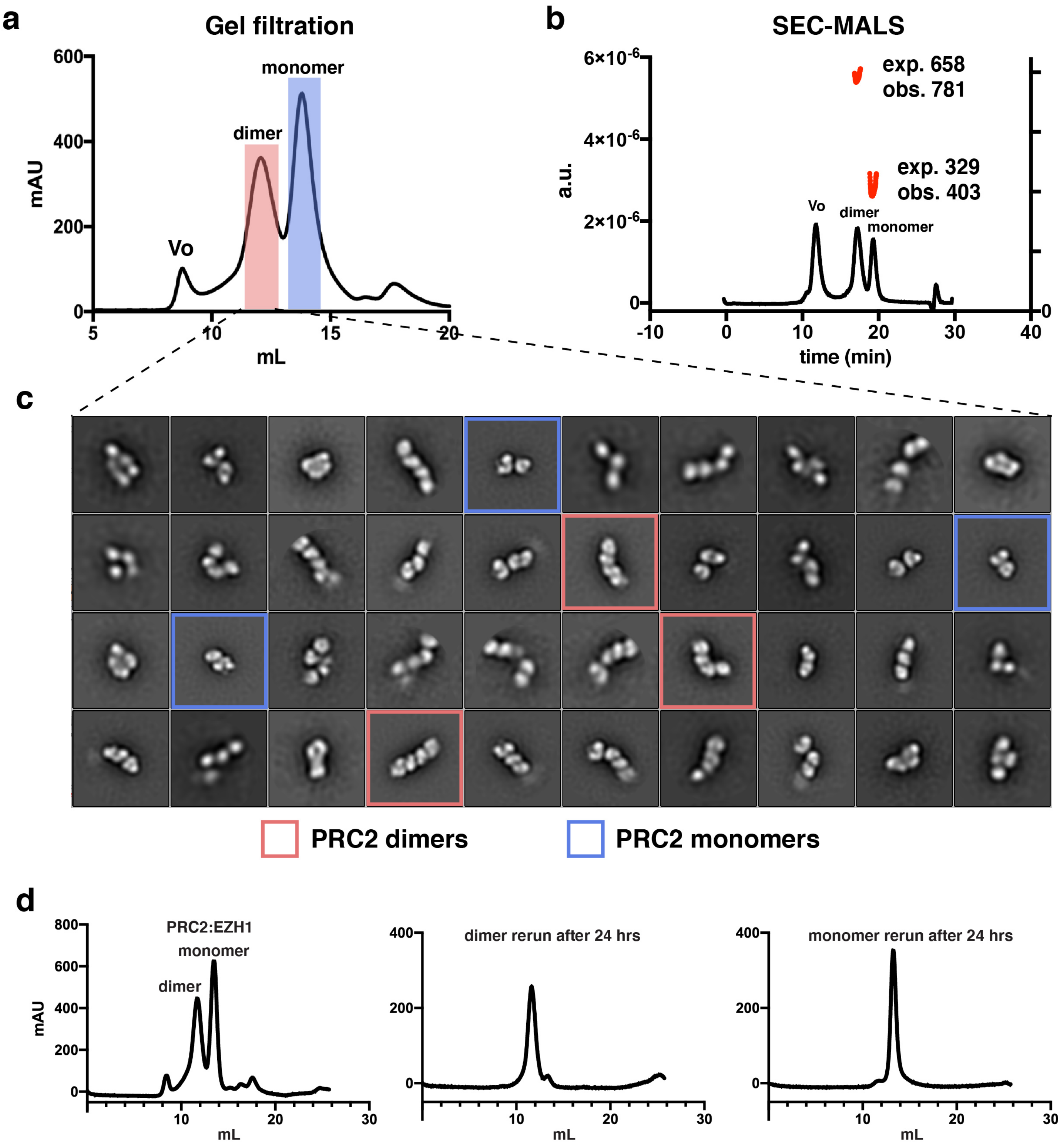

Supplementary Fig. 10: Biochemical activity of PRC2:EZH1 monomers and dimers.

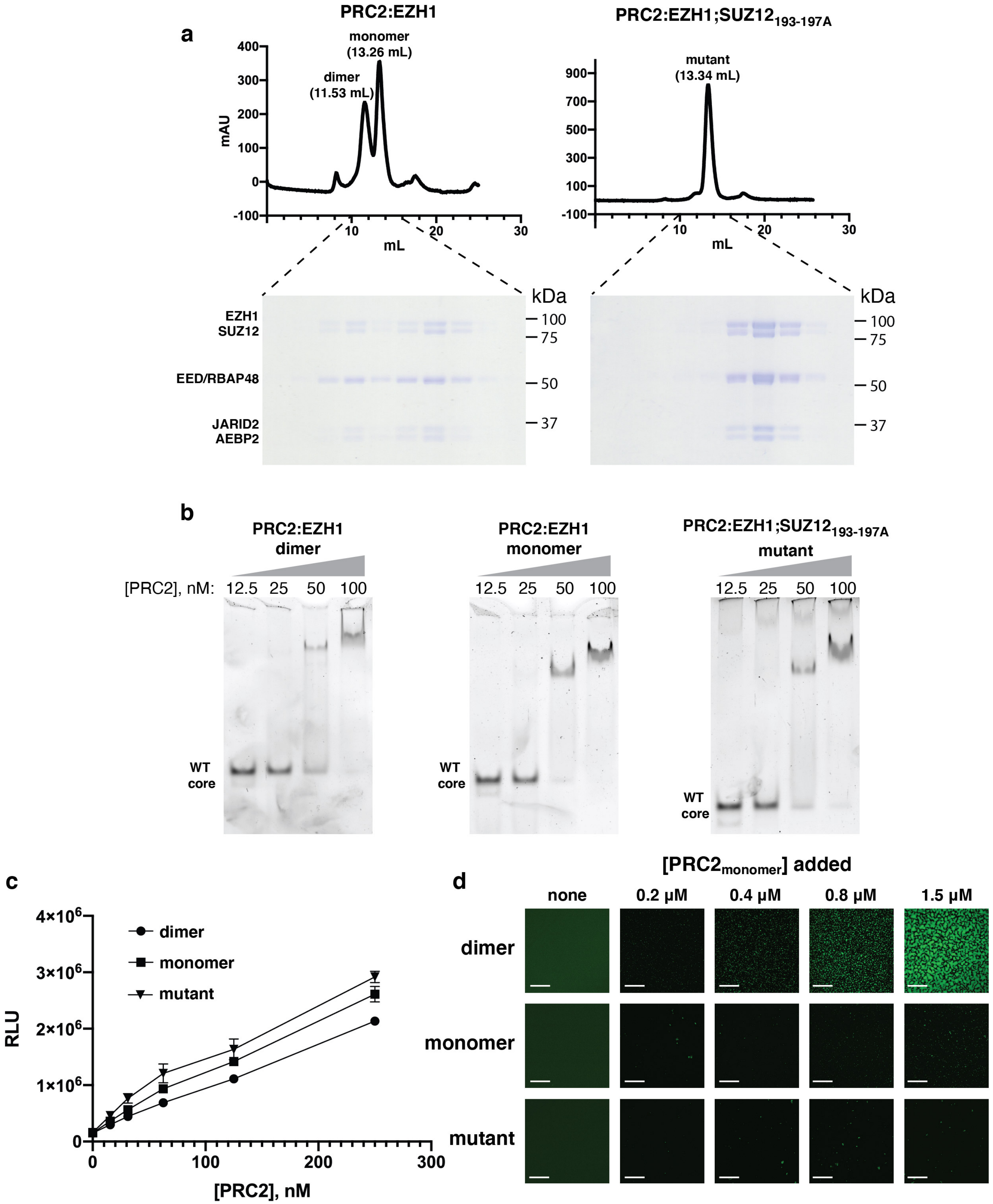

Supplementary Fig. 11: Comparison of PRC2:EZH1/2 dimers.

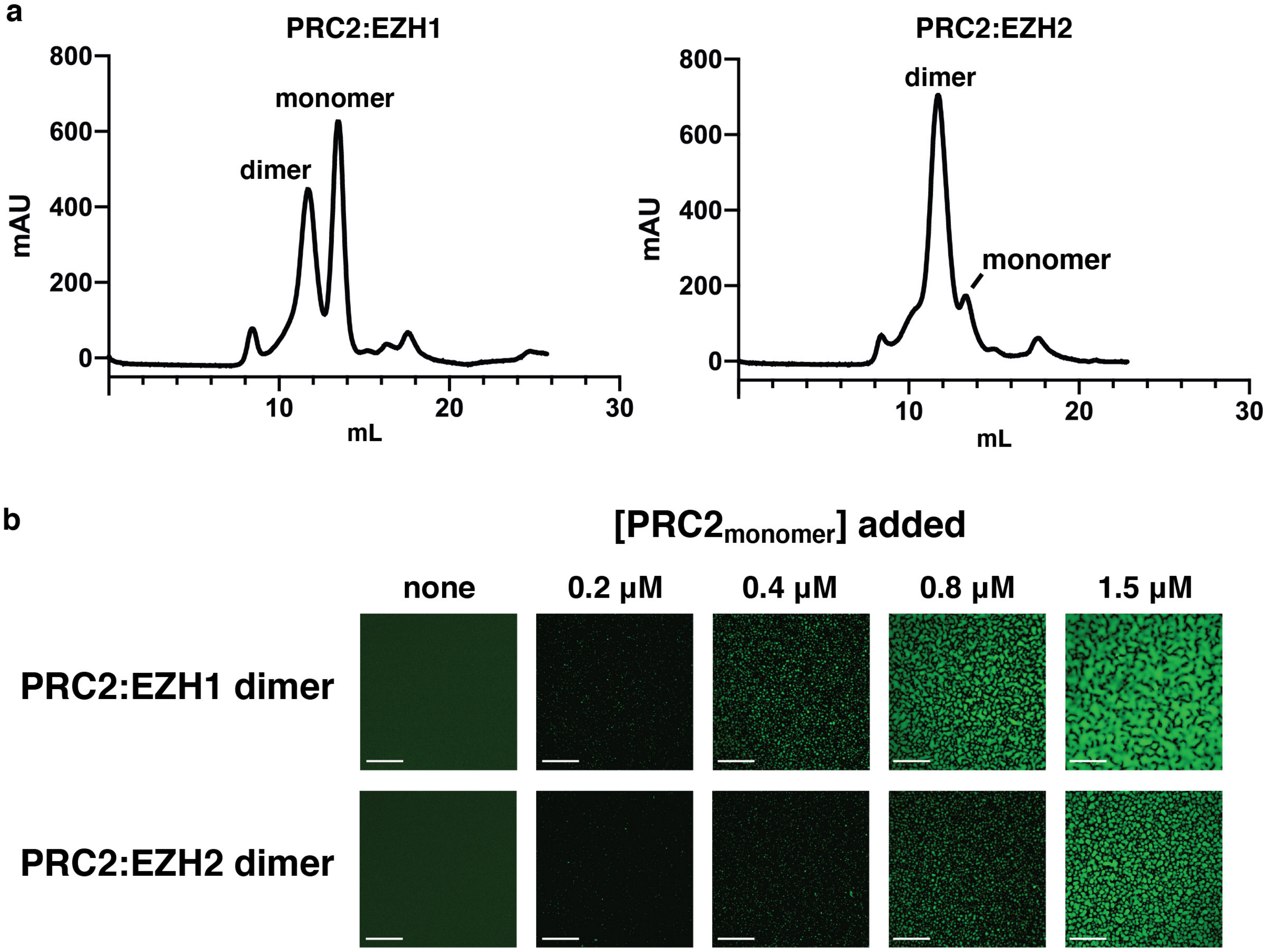

Supplement: Supplementary file 1 — Supplementary Information [file 41467_2020_20775_MOESM1_ESM.pdf]
