# Supplementary material for: Structures of monomeric and dimeric PRC2:EZH1 reveal flexible modules involved in chromatin compaction
Source: Nat Commun. 2021 Jan 29;12:714. doi: 10.1038/s41467-020-20775-z (PMC7846606; doi:10.1038/s41467-020-20775-z)
Supplement: Supplementary file 2 — Reporting Summary [file 41467_2020_20775_MOESM2_ESM.pdf]

nature research

Corresponding author(s): Armache, KJ and Walz, T

Last updated by author(s): Dec 12, 2020

Reporting Summary

Nature Research wishes to improve the reproducibility of the work that we publish. This form provides structure for consistency and transparency in reporting. For further information on Nature Research policies, see Authors & Referees and the Editorial Policy Checklist .

Statistics

For all statistical analyses, confirm that the following items are present in the figure legend, table legend, main text, or Methods section.

n/a	Confirmed	
☐	☒	The exact sample size (*n*) for each experimental group/condition, given as a discrete number and unit of measurement
☒	☐	A statement on whether measurements were taken from distinct samples or whether the same sample was measured repeatedly
☒	☐	The statistical test(s) used AND whether they are one- or two-sided<br>*Only common tests should be described solely by name; describe more complex techniques in the Methods section.*
☒	☐	A description of all covariates tested
☒	☐	A description of any assumptions or corrections, such as tests of normality and adjustment for multiple comparisons
☐	☒	A full description of the statistical parameters including central tendency (e.g. means) or other basic estimates (e.g. regression coefficient) AND variation (e.g. standard deviation) or associated estimates of uncertainty (e.g. confidence intervals)
☒	☐	For null hypothesis testing, the test statistic (e.g. F, t, r) with confidence intervals, effect sizes, degrees of freedom and P value noted<br>*Give P values as exact values whenever suitable.*
☒	☐	For Bayesian analysis, information on the choice of priors and Markov chain Monte Carlo settings
☒	☐	For hierarchical and complex designs, identification of the appropriate level for tests and full reporting of outcomes
☒	☐	Estimates of effect sizes (e.g. Cohen's *d*, Pearson's *r*), indicating how they were calculated

Our web collection on statistics for biologists contains articles on many of the points above.

Software and code

Policy information about availability of computer code

Data collection	Cryo-EM data collection: SerialEM v3.8, Typhoon scanner control v5.0, Zen v2.5.
Data analysis	Cryo-EM analysis: MotionCorr2, Ctffind4, Gautomatch, cryoSPARC v2, Relion v2.1, 3.0, 3,1, Chimera v1.14, ChimeraX v1.0, PyMol v2, Phenix v1, Coot v0.8, ImageJ v2, Prism v8.

For manuscripts utilizing custom algorithms or software that are central to the research but not yet described in published literature, software must be made available to editors/reviewers. We strongly encourage code deposition in a community repository (e.g. GitHub). See the Nature Research guidelines for submitting code & software for further information.

Data

Policy information about availability of data

All manuscripts must include a data availability statement. This statement should provide the following information, where applicable:

- Accession codes, unique identifiers, or web links for publicly available datasets
- A list of figures that have associated raw data
- A description of any restrictions on data availability

Please contact karim-jean.armache@nyulangone.org for any inquiries or material requests. All data are available in the main text, or supplementary materials.

Data availability ----please add the composite map
Data supporting the findings of this manuscript are available from the corresponding authors upon reasonable request. A reporting summary for this article is available as a Supplementary Information file. The Cryo-EM density maps of the PRC2:EZH1–AEBP2: EMD-23022, PRC2:EZH1–AEBP2–JARID2: EMD-23021, PRC2–nucleosome (nucleosome): EMD-23026, PRC2–nucleosome (PRC2_A): EMD-23024, PRC2–nucleosome (PRC2_B): EMD-23025 and PRC2–nucleosome (composite map): EMD-23103 complexes have been deposited in the Electron Microscopy Data Bank. The atomic coordinates for PRC2:EZH1–AEBP2–JARID2: 7KSO, PRC2–nucleosome (nucleosome): 7KTQ, PRC2–nucleosome (PRC2_A): 7KSR, and PRC2–nucleosome (PRC2_B): 7KTP complexes have been deposited in the RCSB Protein Data Bank. The source data underlying Figs. 2c,d,e,f, 7b, and 10c are provided as a Source Data file.

Field-specific reporting

Please select the one below that is the best fit for your research. If you are not sure, read the appropriate sections before making your selection.

[x] Life sciences [ ] Behavioural & social sciences [ ] Ecological, evolutionary & environmental sciences

For a reference copy of the document with all sections, see nature.com/documents/nr-reporting-summary-flat.pdf

Life sciences study design

All studies must disclose on these points even when the disclosure is negative.

Sample size	No sample sizes were predetermined. For in vitro experiments, three replicates were done to determine experimental variation. Where applicable, biological replicates were tested with similar results.
Data exclusions	No data were excluded.
Replication	Experiments where data are graphed are the result of three independent replicates. All experiments were done at least twice with biological replicates and displayed similar results.
Randomization	Randomization is not relevant to this study as statistical tests were not used in the overall interpretations and conclusions of the paper.
Blinding	Blinding is not relevant to this study as statistical tests were not used in the overall interpretations and conclusions of the paper.

Reporting for specific materials, systems and methods

We require information from authors about some types of materials, experimental systems and methods used in many studies. Here, indicate whether each material, system or method listed is relevant to your study. If you are not sure if a list item applies to your research, read the appropriate section before selecting a response.

Materials & experimental systems

n/a	Involved in the study	
[x]	[ ]	Antibodies
[ ]	[x]	Eukaryotic cell lines
[x]	[ ]	Palaeontology
[x]	[ ]	Animals and other organisms
[x]	[ ]	Human research participants
[x]	[ ]	Clinical data

Methods

n/a	Involved in the study	
[x]	[ ]	ChIP-seq
[x]	[ ]	Flow cytometry
[x]	[ ]	MRI-based neuroimaging

Eukaryotic cell lines

Policy information about cell lines

Cell line source(s)	Spodoptera frugiperda (Sf9): ATCC
Authentication	Cell line was authenticated by morphology and growth characteristics.
Mycoplasma contamination	Sf9 cells were not tested for mycoplasma
Commonly misidentified lines (See ICLAC register)	N/A
