# Supplementary material for: Structures of monomeric and dimeric PRC2:EZH1 reveal flexible modules involved in chromatin compaction
Source: Nat Commun. 2021 Jan 29;12:714. doi: 10.1038/s41467-020-20775-z (PMC7846606; doi:10.1038/s41467-020-20775-z)
Supplement: Supplementary file 3 — Description of Additional Supplementary Files [file 41467_2020_20775_MOESM3_ESM.pdf]

Description of Additional Supplementary Files

Supplementary Movie 1: Major structural differences between models of monomeric and dimeric nucleosome-bound forms of PRC2:EZH1.
Model of PRC2:EZH1 monomer morphing to the dimeric PRC2:EZH1 bound to a nucleosome. Rotations of the lower lobe of PRC2:EZH1 and the SUZ12 C2 domain are shown. Rotations represent morphing of the two final static models and do not imply actual movements.
